# Differential Afa/Dr Fimbriae Expression in the Multidrug-Resistant Escherichia coli ST131 Clone

**DOI:** 10.1128/mbio.03519-21

**Published:** 2022-01-18

**Authors:** Laura Alvarez-Fraga, Minh-Duy Phan, Kelvin G. K. Goh, Nguyen Thi Khanh Nhu, Steven J. Hancock, Luke P. Allsopp, Kate M. Peters, Brian M. Forde, Leah W. Roberts, Matthew J. Sullivan, Makrina Totsika, Scott A. Beatson, Glen C. Ulett, Mark A. Schembri

**Affiliations:** a School of Chemistry and Molecular Biosciences, The University of Queensland, Brisbane, Queensland, Australia; b Australian Infectious Diseases Research Centre, The University of Queensland, Brisbane, Queensland, Australia; c University Hospital Complex of A Coruña (CHUAC), Biomedical Research Institute of A Coruña (INIBIC), A Coruña, Spain; d School of Pharmacy and Medical Sciences, and Menzies Health Institute Queensland, Griffith University, Southport, Queensland, Australia; e National Heart and Lung Institute, Imperial College London, London, UK; f University of Queensland Centre for Clinical Research, Brisbane, Queensland, Australia; g EMBL-EBI, Wellcome Genome Campus, Hinxton, Cambridgeshire, UK; h Centre for Immunology and Infection Control, School of Biomedical Sciences, Queensland University of Technology, Herston, Queensland, Australia; The Ohio State University School of Medicine

**Keywords:** adhesins, antibiotic resistance, fimbriae, uropathogenic *Escherichia coli*, virulence regulation

## Abstract

Many antibiotic resistant uropathogenic Escherichia coli (UPEC) strains belong to clones defined by their multilocus sequence type (ST), with ST131 being the most dominant. Although we have a good understanding of resistance development to fluoroquinolones and third-generation cephalosporins by ST131, our understanding of the virulence repertoire that has contributed to its global dissemination is limited. Here we show that the genes encoding Afa/Dr fimbriae, a group of adhesins strongly associated with UPEC that cause gestational pyelonephritis and recurrent cystitis, are found in approximately one third of all ST131 strains. Sequence comparison of the AfaE adhesin protein revealed a unique allelic variant carried by 82.9% of *afa*-positive ST131 strains. We identify the *afa* regulatory region as a hotspot for the integration of insertion sequence (IS) elements, all but one of which alter *afa* transcription. Close investigation demonstrated that the integration of an IS*1* element in the *afa* regulatory region leads to increased expression of Afa/Dr fimbriae, promoting enhanced adhesion to kidney epithelial cells and suggesting a mechanism for altered virulence. Finally, we provide evidence for a more widespread impact of IS*1* on ST131 genome evolution, suggesting that IS dynamics contribute to strain level microevolution that impacts ST131 fitness.

## INTRODUCTION

Uropathogenic Escherichia coli (UPEC) is the most common cause of urinary tract infections (UTIs) and a frequent cause of life-threatening sepsis (1). The majority of UPEC strains belong to global clones that can be differentiated based on their multilocus sequence type (ST), including ST69, ST73, ST95, and ST131 (2–4). ST131 is the most globally dominant high-risk UPEC clone and a major contributor to increasing antibiotic resistance (2, 5). ST131 was first identified in 2008 and is associated with resistance to multiple antibiotic classes, including fluoroquinolones, third-generation cephalosporins and more recently last-line carbapenems and polymyxins (6–9).

While genomic studies have accurately traced the epidemiology and emergence of ST131 (7, 10–12), the precise molecular mechanisms that have led to the dominance of ST131 over other UPEC clones remain unclear. ST131 shares many common virulence factors with non-ST131 UPEC, including adhesins, toxins, capsule polysaccharides, and iron acquisition systems. The Dr family or Afa/Dr chaperone-usher fimbriae represent one important UPEC virulence determinant that have been suggested to display a higher prevalence in ST131 compared with non-ST131 UPEC (4, 13). Afa/Dr fimbriae are frequently associated with UPEC that cause cystitis in children, as well as pyelonephritis and recurrent UTIs in young and pregnant women (14–18). The receptors for Afa/Dr fimbriae are the Dr blood group antigen on the human decay-accelerating factor (DAF), some members of the carcinoembryonic antigen (CEA) family and type IV collagen (18–21). Interactions between Afa/Dr fimbriae and its host receptor stimulate invasion, leading to the avoidance of host immunosurveillance and antibiotic treatment (22–24), and possibly contributing to recurrent infection (14, 18).

Members of the Dr family were initially reported to form both fimbrial and afimbrial structures on the bacterial surface (14, 25–28). However, more recent studies have revealed the afimbrial pattern results from the collapse of flexible fimbrial structures onto the bacterial surface, demonstrating they are indeed assembled as fimbrial organelles (29, 30). Afa/Dr fimbriae are closely related at the DNA level, share a similar genetic organization, and have been described using a range of gene designations including *dra*, *drb*, *nfa*, *agg*, *hda*, and *afa* (used here) (31, 32). Afa/Dr fimbriae are encoded by a cluster of genes organized in two divergent transcriptional units - a primary transcriptional unit comprising genes encoding a regulator (e.g., *afaA*), chaperone (e.g., *afaB*), usher (e.g., *afaC*), repeating major adhesin subunit (e.g., *afaE*), and tip-associated capping subunit protein (e.g., a*faD*), as well as a minor transcriptional unit comprising a single regulatory gene (e.g., *afaF*) (14, 33, 34). The AfaE major adhesin subunit exhibits extensive amino acid sequence diversity and is under positive selection pressure that leads to altered binding phenotypes, including increased binding to the DAF receptor (35). Afa/Dr expression is phase variable due to the concerted action of deoxyadenosine methylase (Dam) and the leucine-responsive regulatory protein (Lrp) that control differential methylation patterns in the *afa/dr* promoter region (36, 37), as well as activation by integration host factor (IHF) and repression by the histone-like nucleoid-structuring protein H-NS (37, 38). The *afa/dr* genes have been shown to be located on plasmids or within chromosomal genomic islands (GIs) (39, 40), supporting their capacity to be transferred via mobile genetic elements. Furthermore, the *afa-3* allelic variant present in the UPEC cystitis isolate A30 is flanked by IS*1* elements that facilitate plasmid to chromosome transfer via IS*1*-mediated recombination (33, 41).

Previous *in vivo* studies have demonstrated the role of Afa/Dr adhesins in UPEC virulence. The *draE* and *afaE-III* alleles are among the most widely studied and have been associated with chronic tubulointerstitial nephritis (42) and mortality of pregnant rats (43). In this study, we examined the distribution of *afa/dr* genes among the most common E. coli STs, confirming their high prevalence in ST131. Close examination of the *afa/dr* gene complexity in ST131 showed the regulatory region is a hotspot for the integration of IS elements, and we demonstrate that IS*1* insertion leads to increased Afa fimbrial expression, highlighting the capacity of IS elements to drive altered virulence at the strain level. Finally, we expand these findings by mapping the integration of IS*1* within the ST131 pangenome, thus revealing how the widespread occurrence of this mobile genetic element can impact ST131 genome evolution.

## RESULTS

### Afa/Dr fimbrial genes are highly prevalent in E. coli ST131.

The prevalence of Afa/Dr fimbrial genes in E. coli was initially examined by investigating their distribution in genomes representing the most common STs from EnteroBase, a large publicly available *Enterobacteriaceae* genome sequence database (44). Analysis of 100 randomly selected genome assemblies from each of the 83 highest-represented STs revealed the *afaC* usher gene, which is conserved in all *afa/dr* loci, is present in 18 different STs. The highest incidence was found in ST131 (35%; 35/100) and ST38 (37%; 37/100); followed by ST59 (17%; 17/100), ST405 (14%; 14/100), ST448 (12%; 12/100), and ST648 (12%; 12/100) (Fig. S1A in the supplemental material). Overall, the Afa/Dr fimbrial genes were found in STs from all phylogroups, but at different prevalence rates.

10.1128/mbio.03519-21.1 FIG S1 Distribution of Dr family fimbriae in (A) strains belonging to the 83 most common E. coli STs and (B) strains belonging to the five most dominant UPEC clones. E. coli STs are grouped into seven main phylogenetic groups: A (ST10, ST34, ST48, ST93, ST167, ST301, and ST746), B1 (ST448), B2 (ST12 and ST131), C (ST23 and ST410), D (ST38, ST405 and ST69), E (ST335), and F (ST59 and ST648). The percentage of strains containing Afa/Dr fimbriae was determined by tBLASTn against *afaC*^EC958^, with a cut-off value of > 90% nucleotide identity and > 80% gene coverage. Download FIG S1, TIF file, 0.3 MB. Copyright © 2022 Alvarez-Fraga et al. 2022 Alvarez-Fraga et al. https://creativecommons.org/licenses/by/4.0/ This content is distributed under the terms of the Creative Commons Attribution 4.0 International license.

We were particularly interested in the high prevalence of the Afa/Dr fimbrial genes in ST131, and therefore expanded our screen to perform a detailed comparison of ST131 (phylogroup B2) and four other UPEC dominant STs: ST10 (phylogroup A), ST69 (phylogroup D), ST73 (phylogroup B2), and ST95 (phylogroup B2). This large-scale analysis revealed that the prevalence of Afa/Dr fimbrial genes largely mirrored our results from the 83 ST screen, with 29% (1,157/3,993) of ST131 strains containing the *afaC* usher gene, compared with ST10 (65/4,731; 1.4%), ST69 (16/805; 2%), ST73 (2/923; 0.2%), and ST95 (1/831; 0.1%) (Fig. S1B).

**FIG 1:**
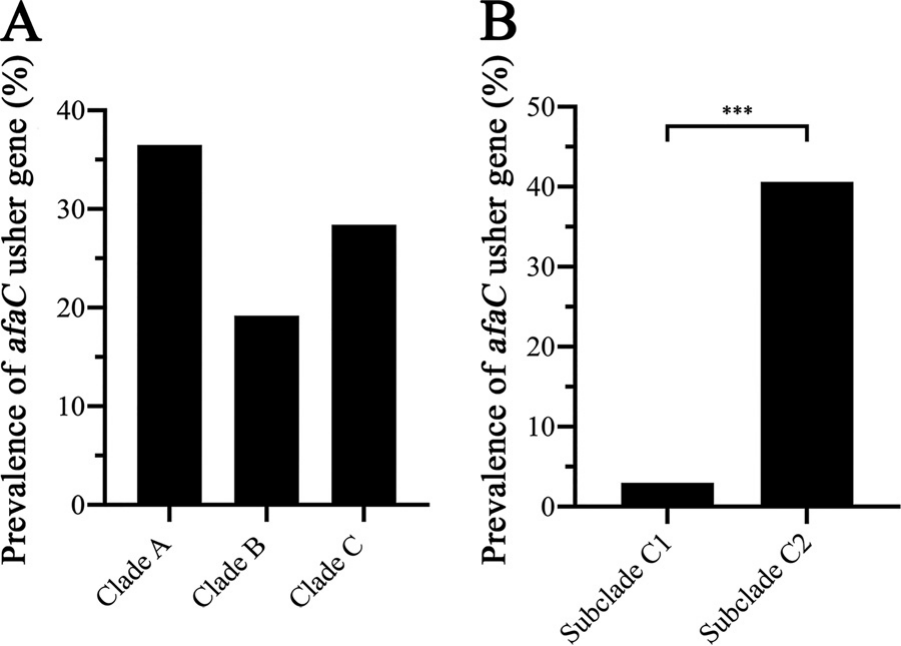
Distribution of Afa/Dr fimbrial genes in strains belonging to the ST131 lineage; (A) clade A, B, and C; and (B) subclade C1 and C2. The percentage of strains containing Afa/Dr fimbriae was determined by tBLASTn against *afaC*^EC958^, with a cut-off value of > 90% nucleotide identity and > 80% gene coverage. ST131 strains were classified in clades and subclades based on clade-specific SNPs. One-hundred and 36 ST131 strains could not be classified into any clade, and thus were excluded from the analysis. Chi-squared test was used for statistical analysis (***, *P < *0.0001 for pair of C2–C1).

Despite the enormous diversity of UPEC at the genome level, the ST131 lineage represents a monophyletic clone with a well-defined genealogy (7, 10, 12, 45, 46). ST131 is comprised of three major sublineages, clades A, B, and the fluoroquinolone-resistant clade C. A total of 3,857 ST131 strains from the Enterobase data set were categorized into their specific sublineage using clade-defining single nucleotide polymorphisms (SNPs) that we have characterized previously (10). One-hundred and 36 strains could not be classified into any clade, and were excluded from this analysis. Overall, 36.5% (138/378) of clade A strains, 19.2% (66/344) of clade B strains, and 28.4% (890/3,135) of clade C strains possessed the *afaC* usher gene (Fig. 1A). Within clade C, the prevalence of the Afa/Dr fimbrial genes was significantly more common in strains from the multidrug-resistant subclade C2 (40.6%; 840/2,069) compared with subclade C1 (3%; 30/993) (*P < *0.0001; Chi-square test) (Fig. 1B).

### Variation of the AfaE major subunit protein in ST131 is dominated by a unique variant.

**FIG 2:**
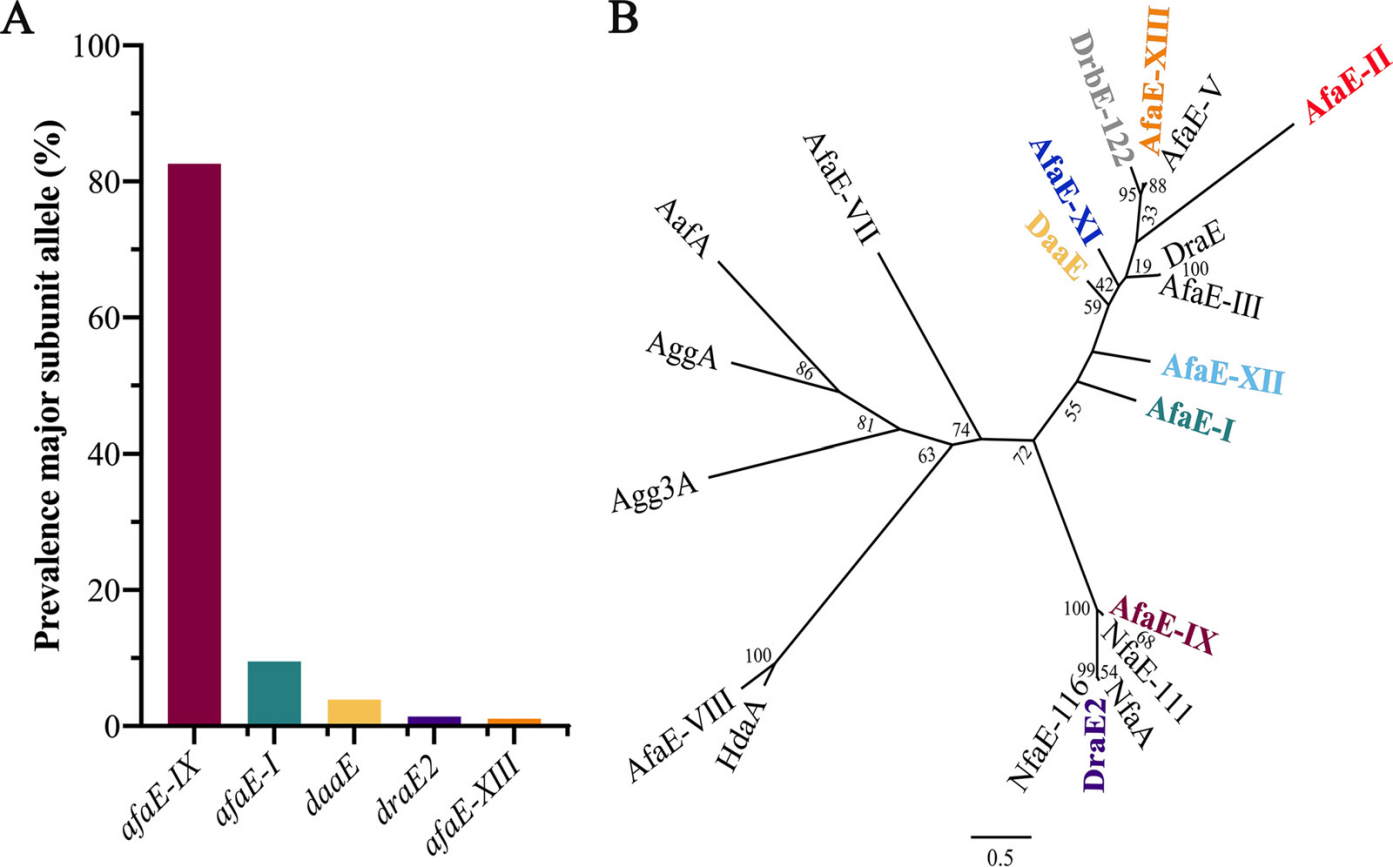
(A) Prevalence of the major subunit allelic variants found in the ST131 lineage. The percentage of each *afaE* allele was determined by tBLASTn with a cut-off value of > 97% nucleotide identity and > 80% gene coverage. The *afaE-XI*, *afaE-II*, *afaE-XII*, and *drbE-122* alleles were prevalent at < 1% (not shown). (B) Unrooted ML phylogenetic tree showing the relationship between the AfaE variants. The names in color represent the AfaE variants found in the ST131 lineage. The scale indicates the number of amino acid substitutions; numbers refer to the bootstrap values.

Analysis of the nucleotide sequence of the Afa/Dr major subunit gene in our ST131 data set identified nine allelic variants (Fig. 2A). Among these, 82.9% (907/1,094) of the strains possessed an identical *afaE* allele, which was the same as the one found in the reference ST131 EC958 genome (referred to as *afaE-IX)*. Other *afaE* alleles were present at lower levels: *afaE-I* (9.6%; 105/1,094), *daaE* (3.7%; 41/1,094), *draE2* (1.4%; 15/1,094), *afaE-II* (0.3%; 3/1,094), *drbE-122* (0.1%; 1/1,094), and three previously unreported *afaE* alleles (referred to as *afaE-XIII*; 1.1%; 12/1,094), *afaE-XI* (0.7%; 8/1,094), and *afaE-XII* (0.2%; 2/1094) (Fig. 2A). The *afaE-IX* allele was predominant in all ST131 clades, and found at a significantly higher rate in subclade C2 (37.5%) compared with C1 (1.6%) (*P < *0.0001; Chi-square test) (Fig. S2A, B).

10.1128/mbio.03519-21.2 FIG S2 (A) Prevalence of the major subunits allelic variants in ST131 clades. AfaE alleles *afaE-IX, afaE-I, daaE* and *draE2* are shown; five alleles were prevalent at < 1% (not shown): *afaE-XIII* (0.4%, clade C)*, afaE-XI* (0.2%, clade B and 0.2%, clade C)*, afaE-II* (0.1%, clade C)*, afaE-XII* (0.1%, clade C), and *drbE-122* (0.03%, clade C). (B) Prevalence of the major subunits allelic variants in ST131 subclades C1 and C2. AfaE alleles *afaE-IX* and *afaE-I* are shown; four alleles were prevalent at < 1% (not shown): *afaE-XIII* (0.5%, subclade C1 and 0.3%, subclade C2)*, drbE-122* (0.3%, subclade C1)*, afaE-XI* (0.3%, subclade C2)*, afaE-II* (0.1%, subclade C2)*, afaE-XII* (0.2%, subclade C2). The *daaE* and *afaE-XIV* alleles were absent in both subclades. The percentage of each *afaE* allele was determined by tBLASTn with a cut-off value of > 97% nucleotide identity and > 80% gene coverage. ST131 strains were classified in clades and subclades based on clade-specific SNPs. One-hundred and 36 ST131 strains could not be classified into any clade and thus were excluded from the analysis. Chi-squared test was used for statistical analysis (*** *P < *0.0001 for pair of C2–C1). (C) Prevalence of the major subunit allelic variants within E. coli ST38 clone. The *daaE*, *afaE-XII*, and *afaE-VIII* alleles were prevalent at < 1% (not shown). The percentage of each *afaE* allele was determined by tBLASTn with a cut-off value of > 97% nucleotide identity and > 80% gene coverage. Download FIG S2, TIF file, 0.5 MB. Copyright © 2022 Alvarez-Fraga et al. 2022 Alvarez-Fraga et al. https://creativecommons.org/licenses/by/4.0/ This content is distributed under the terms of the Creative Commons Attribution 4.0 International license.

Due to the high prevalence of Afa/Dr fimbrial genes in the subset of 100 ST38 genomes, we also downloaded all 1,926 ST38 genomes from EnteroBase and analyzed their overall prevalence and *afaE* allelic variation. The *afaC* usher gene was present in 38.6% (743/1,926) of the ST38 strains. The most prevalent major subunit alleles were *afaE-XI* (33%; 245/743), *drbE-122* (21.4%; 159/743), *afaE-II* (15.7%; 117/743), and *draE2* (14.8%; 110/743). Other *afaE* alleles were present at lower levels: *afaE-I* (5.8%; 43/743), *afaE-IX* (6.1%; 45/743), *afaE-XIII* (1.3%; 10/743), *daaE* (0.9%; 7/743), *afaE-XII* (0.8%; 3/743), and *afaE-VIII* (0.1%, 1/743) (Fig. S2C). We were unable to identify the major subunit allele of three strains. Thus, although both ST131 and ST38 possess a high prevalence of Afa/Dr fimbrial genes, the distribution of *afaE* allelic variants differs markedly within each clone.

The phylogenetic relationship between all identified AfaE protein variants was examined by generating a multiple-sequence alignment of the mature proteins (Fig. S3). The ST131 AfaE variants could be assigned to previously described distinct phylogenetic groups (35). The dominant AfaE-IX variant forms a discrete group together with the NfaE-111 variant (Fig. 2B). The AfaE-IX and NfaE-111 proteins share 96.2% identity, with all differences centered around one of the predicted DAF binding sides (19, 30, 47) (Fig. S3). The novel AfaE-XIII variant also forms a distinct group, together with AfaE-V (Fig. 2B). Finally, the AfaE-XI and AfaE-XII variants form two distantly related groups (Fig. 2B).

10.1128/mbio.03519-21.3 FIG S3 Multiple-sequence alignment of the major subunit variants. The sequences correspond to the mature protein. Each rectangle represents a distinct group, and their phylogenetic relationship is shown in the cladogram. The rectangles in color represent the groups found in the ST131 lineage. The predicted CEA and DAF binding sites are boxed in the protein sequence. The AfaE-XI variant shares a 99.6% identity with the AfaE variant found in the strain VR50. Download FIG S3, TIF file, 2.9 MB. Copyright © 2022 Alvarez-Fraga et al. 2022 Alvarez-Fraga et al. https://creativecommons.org/licenses/by/4.0/ This content is distributed under the terms of the Creative Commons Attribution 4.0 International license.

### The *afa*/*dr* regulatory region is a hotspot for IS integration.

**FIG 3:**
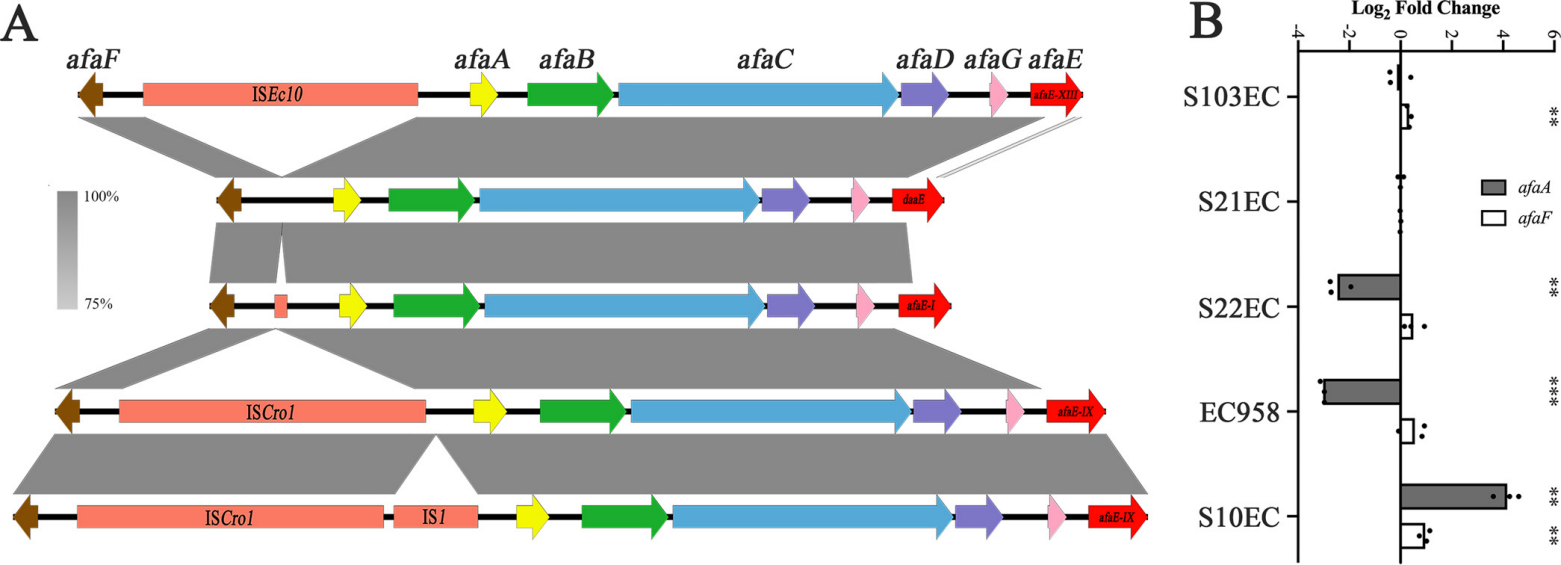
(A) Schematic representation of the Afa/Dr fimbrial gene clusters found in ST131, showing the IS elements located in the promoter region. Brown (*afaF*) and yellow (*afaA*) arrows represent genes encoding transcriptional regulators; orange rectangles represent different IS elements; other genes are color-coded as green (chaperone), blue (usher), purple (invasin), pink (genes involved in RNA processing), and red (adhesin major subunit). (B) Transcript levels of *afaA* and *afaF* genes in representative ST131 strains. The results are represented as log_2_ fold change compared with S21EC (which has no IS element in the promoter region). The *gapA* gene was used as an endogenous control. Experiments were performed in three biological replicates. Unpaired Student's *t* test was used for statistical analysis (**, *P* value < 0.01; ***, *P* value < 0.0001).

Closer examination of the *afa*/*dr* gene cluster in our ST131 genome data set revealed the presence of IS elements in the regulatory region located between the divergently transcribed *afaF* and *afaA* genes (Fig. 3A). All strains containing the *afaE-IX* allele possessed a large 2,824-bp IS*Cro1* element (IS*66* family) 440 bp upstream of the *afaA* start codon. Five of these strains also possessed an IS*1* element 87 bp downstream of the IS*Cro1* element. The genomic insertion site of IS*Cro1* appears to be a hotspot for IS integration, as strains possessing other *afaE* adhesin alleles contain different insertions at this site; strains containing the *afaE-I* allele possess a 118-bp insertion of unknown origin, while strains containing the *afaE-XIII* allele possess a 2,530-bp IS*Ec10* (IS*21* family) insertion at this site. Finally, strains containing the *daaE*, *draE2*, and *afaE-XI* alleles did not have any insertions in the *afa/dr* promoter region (Fig. 3A). We were unable to assemble the *afa*/*dr* promoter region associated with the *afaE-XIV*, *afaE-XII*, and *afaE-II* alleles, and thus could not accurately examine the presence of IS elements in strains containing these variants.

To determine the precise genetic location of *afa/dr* genes representing each *afaE* allelic variant, we generated complete genome sequences of representative strains: S21EC (*daaE*; control strain with no insertion), S22EC (*afaE-I*/118-bp insertion), S10EC (*afaE-IX*/IS*Cro1*-IS*1*), and S103EC (*afaE-XIII*/IS*Ec10*). Summary features of these genomes, including chromosome size and plasmids are presented in Data set S1A; antibiotic resistance genes and major virulence factors were described previously based on analyses of draft genomes (7). The complete genome of EC958 (*afaE-IX*/IS*Cro1*) has been published previously (48). Analysis of the genome data revealed the *afa/dr* gene cluster is located within a genomic island (GI) integrated at the *pheU*-tRNA in S103EC (clade C2) and S22EC (clade B) (GI-S103EC-*pheU* and GI-S22EC-*pheU*) and *selC*-tRNA in EC958 (clade C2) and S10EC (clade C2) (GI-EC958-*selC* and GI-S10EC-*selC*) (Fig. S4A). In contrast, the *afa/dr* gene cluster in S21EC (clade B) is located within an insertion fragment integrated within the *ulaE* gene (GI-S21EC-*ulaE*) (Fig. S4A). The sequence variation and different genomic location of the *afa/dr* genes suggest they have been acquired independently of their clade designation (Fig. S4B).

10.1128/mbio.03519-21.4 FIG S4 (A) Comparative genomics between the ST131 representative strains. Prophages (Phi) are indicated by blue boxes; GI are indicated by purple boxes and the GI containing the *afa/dr* gene cluster are indicated by green boxes. The *ulaE* gene corresponds to locus tag S21EC_48440. (B) Comparative genomics between the genomic islands carrying the *afa/dr* operon in the ST131 representative strains. Brown (*afaF*) and yellow (*afaA*) arrows represent genes encoding transcriptional regulators; orange arrows represent different IS elements; other genes are color-coded as green (chaperone), blue (usher), purple (invasin), pink (genes involved in RNA processing), and red (adhesin major subunit). Download FIG S4, TIF file, 0.9 MB. Copyright © 2022 Alvarez-Fraga et al. 2022 Alvarez-Fraga et al. https://creativecommons.org/licenses/by/4.0/ This content is distributed under the terms of the Creative Commons Attribution 4.0 International license.

10.1128/mbio.03519-21.10 DATA SET S1 (A) Summary features of ST131 representative strains. (B) Chromosomal IS*1* insertion sites found in ST131 clone relative to six complete reference genomes. (C) Number of IS*1* insertion sites relative to six ST131 complete reference genomes. (D) Chromosomal IS*1* insertion sites found in ST131 clone relative to EC958 reference genome. (E) Primers used in this study. Download Data Set S1, XLSX file, 0.2 MB. Copyright © 2022 Alvarez-Fraga et al. 2022 Alvarez-Fraga et al. https://creativecommons.org/licenses/by/4.0/ This content is distributed under the terms of the Creative Commons Attribution 4.0 International license.

### IS integration alters the transcription of *afa/dr* genes.

The transposition of IS into non-coding sequences in a bacterial genome can generate several outcomes, including increased transcription of adjacent genes due to the introduction/creation of a strong promoter (49, 50). To investigate the impact of the IS on the transcription of *afa* genes in ST131, we examined the transcript levels of the *afaA* and *afaF* genes in our representative strain set. Compared with S21EC, the transcript level of *afaA* was reduced in the strains possessing the IS*Cro1* (2.5- log_2_ fold change, *P = *0.0008) and the 118-bp segment (3-log_2_ fold change, *P < *0.0001), while the presence of IS*Ec10* had no effect on *afaA* transcription (*P = *0.6286) (Fig. 3B). In contrast, the insertion of IS*1* downstream of the IS*Cro1* in S10EC led to a significant increase in *afaA* transcript level compared to S21EC (4.2-log_2_ fold change, *P = *0.0002) (Fig. 3B). Analysis of the *afaF* transcript level revealed a small increase in the strains possessing the IS*Ec10* (1.3-fold increase, *P = *0.0034) and IS*1* (2-fold increase, *P = *0.0037) compared with the level of *afaF* transcript in S21EC (Fig. 3B).

**FIG 4:**
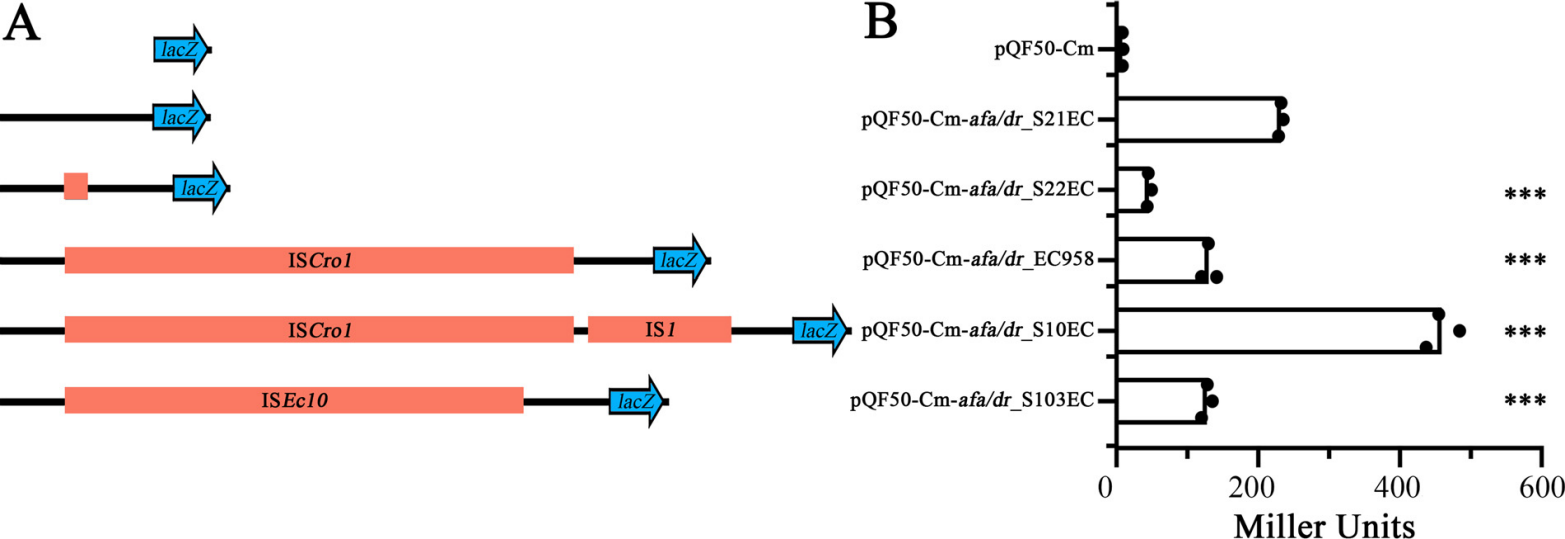
(A) Schematic representation of the *afa/dr* promoter-*lacZ* fusion constructs used in this study. Shown are the different promoter regions cloned immediately upstream of the promoterless *lacZ* gene in pQF50-Cm. The black lines represent the promoter sequence, and the orange rectangles represent the insertion elements. (B) β-galactosidase assay showing the activity of the different *afa/dr* promoters. β-galactosidase activity is expressed as Miller units. Experiments were performed in three biological replicates. Unpaired Student's *t* test was used for statistical analysis (***, *P* value < 0.0001).

Next, we investigated how the different IS elements impact *afa/dr* gene transcription by generating a series of promoter-*lacZ* fusion constructs containing the promoter region from each representative strain cloned into the promoterless *lacZ* vector pQF50-Cm (Table 1). These plasmids were transformed into EC958Δ*lac* and β-galactosidase levels were measured to assess promoter activity (Fig. 4A). The results were largely congruent with the transcript analyses, with the integration of IS*1* resulting in a 2-fold increase in β-galactosidase activity compared to the native promoter (pQF50-Cm-*afa/dr*_S21EC) and a 3.5-fold increase compared to the IS*Cro1*-promoter (pQF50-Cm-*afa/dr*_EC958) (Fig. 4B). In contrast, integration of the 118-bp insertion (pQF50-Cm-*afa/dr*_S22EC), IS*Ec10* (pQF50-Cm-*afa/dr*_S103EC), and IS*Cro1* (pQF50-Cm-*afa/dr*_EC958) significantly reduced β-galactosidase activity compared to the native promoter (pQF50-Cm-*afa/dr*_S21EC) (Fig. 4B). No significant β-galactosidase activity was measured in the strain carrying the empty control plasmid pQF50-Cm.

**TABLE 1:** Bacterial strains and plasmids used in this work

Strain or plasmid	Relevant characteristics	Sources or references
**STRAINS**		
E. coli TOP10	*F–mcrA Δ(mrr-hsdRMS-mcrBC) Φ80lacZΔM15 ΔlacX74 recA1 araD139 Δ(ara leu) 7697 galU galK rpsL (StrR) endA1 nupG*	Invitrogen
EC958	ST131 reference strain (Clade C2). Presence of an IS*Cro1*insertion sequence in the *afa* promoter region and the *afaE-IX* allelic variant.	(7, 48, 84)
EC958Δ*lac*	EC958*lacI-Z::gfp*	(79)
EC958Δ*hns*	EC958*hns::cm*; Cm^r^	(78)
EC958Δ*ihfA*	EC958*ihfA::cm*; Cm^r^	This study
EC958Δ*afaE*	EC958*afaE::cm*; Cm^r^	This study
EC958Δ*afaC*	EC958*afaC::cm*; Cm^r^	This study
S10EC	ST131 UPEC isolate (Clade C2). Presence of an IS*Cro1* and I*S1* insertion sequences in the *afa* promoter region and the *afaE-IX* allelic variant. Presence of an IS*1* upstream of the *osmB* gene.	(7)
S10ECΔ*lac*	S10EC *lacI-Z::gfp*. Cm^r^ cassette removed.	This study
S10ECΔIS*1*	S10EC IS*1*. Cm^r^ cassette removed	This study
S10ECΔ*IS1*Δ*lac*	S10EC IS*1 lacI-Z::gfp.* Cm^r^ cassette removed	This study
S10ECΔ*hns*	S10EC*hns::cm*; Cm^r^	This study
S10ECΔ*ihfA*	S10EC*ihfA::cm*; Cm^r^	This study
S10ECΔ*afaE*	S10EC*afaE::cm*; Cm^r^	This study
S10ECΔ*afaC*	S10EC*afaC::cm*; Cm^r^	This study
S103EC	ST131 UPEC isolate (Clade C2). Presence of an IS*Ec10* insertion sequence in the *afa* promoter region and the *afaE-XIII* allelic variant.	(7)
S21EC	ST131 UPEC isolate (Clade B). Absence of insertion sequences in the *afa* promoter region and presence of the *daaE* allelic variant.	(7)
S22EC	ST131 UPEC isolate (Clade B). Presence of a 118 bp unknown sequence in the *afa* promoter region and the *afaE-I* allelic variant.	(7)
HVM52	ST131 UPEC isolate (Clade B). Presence of an IS*1* upstream of the *ugd* gene.	(7)
HVM277	ST131 UPEC isolate (Clade B). Presence of an IS*1* upstream of the *ugd* gene.	(7)
HVM2044	ST131 UPEC isolate (Clade B). Presence of an IS*1* upstream of the *ugd* gene.	(7)
S12EC	ST131 UPEC isolate (Clade C2). Presence of an IS*1* upstream of the *afaA* and *osmB* genes.	(7)
**PLASMIDS**		
pKD3	Template plasmid for *cm* gene amplification; Cm^r^	(85)
pKOBEG-Gm	λ-red recombinase expressing plasmid; Gm^r^	(84, 86)
pCP20-Gm	FLP expressing plasmid; Gm^r^	(80)
pQF50-Cm	Promoterless *lacZ* reporter plasmid; Cm^r^	(87)
pQF50-Cm-afa/dr_EC958	*afa* promoter region of EC958 cloned in pQF50-Cm	This study
pQF50-Cm-afa/dr_S10EC	*afa* promoter region of S10EC cloned in pQF50-Cm	This study
pQF50-Cm-afa/dr_S10ECΔIS*1*	pQF50-Cm-afa/dr_S10EC without the IS*1* element	This study
pQF50-Cm-afa/dr_S103EC	*afa* promoter region of S103EC cloned in pQF50-Cm	This study
pQF50-Cm-afa/dr_S21EC	*afa* promoter region of S21EC cloned in pQF50-Cm	This study
pQF50-Cm-afa/dr_S22EC	*afa* promoter region of S22EC cloned in pQF50-Cm	This study

### IS*1* is responsible for the high level of AfaE expression in the S10EC strain.

**FIG 5:**
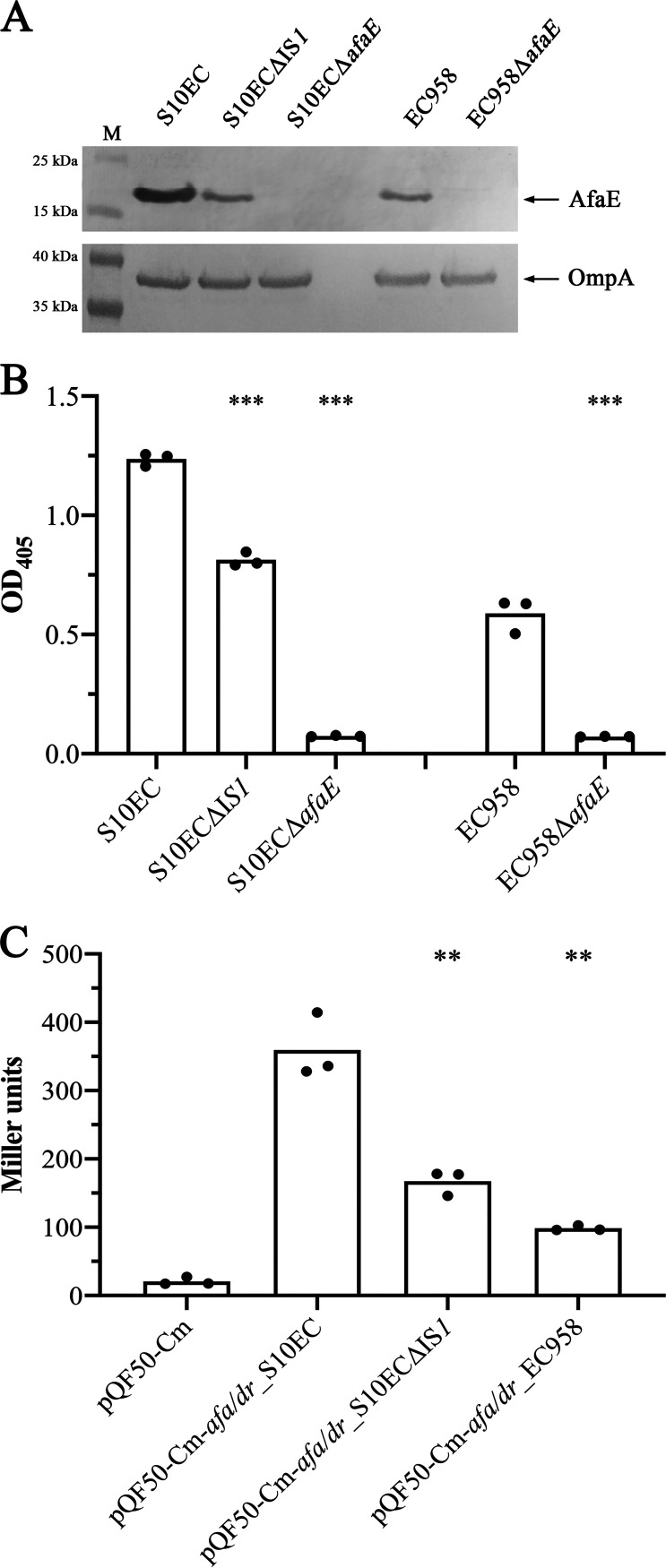
(A) Western blot analysis of AfaE, performed using whole cell lysates prepared from S10EC, S10ECΔIS*1*, S10ECΔ*afaE*, EC958, and EC958Δ*afaE.* Bands corresponding to AfaE (AfaE-IX antibody) and OmpA (loading control, OmpA antibody) are indicated. PageRuler Prestained Protein Ladder (Life Technologies) was used as molecular mass marker (lane M). (B) Graphical presentation of ELISA data showing the level of AfaE protein on the surface of S10EC, S10ECΔIS*1*, S10ECΔ*afaE*, EC958, and EC958Δ*afaE.* The primary polyclonal rabbit anti-AfaE-IX and the secondary alkaline phosphatase-conjugated anti-rabbit antibodies were used. (C) β-galactosidase assay showing the activity of the S10EC, S10ECΔIS*1*, and EC958 *afa/dr* promoters. β-galactosidase activity is expressed as Miller units. Experiments were performed in three biological replicates. Unpaired Student's *t* test was used for statistical analysis (**, *P* value < 0.01; ***, *P < *0.0001).

To further examine the impact of IS*1* integration on Afa expression, the IS*1* element was deleted from the genome of the S10EC strain and the expression of the AfaE major subunit was analyzed by Western blotting using an AfaE-IX specific antibody. Analysis of whole cell lysates prepared from S10EC and EC958 revealed increased expression of AfaE in S10EC compared with EC958. When the IS*1* element was deleted in S10EC, the expression of AfaE was reduced to a level similar to that observed for EC958 (Fig. 5A). No expression of AfaE was detected in the S10ECΔ*afaE* and EC958Δ*afaE* mutant strains used as negative controls. We also examined cell-surface expression of AfaE using a whole-cell ELISA in combination with our AfaE-IX antibody. These analyses revealed a 2.1-fold higher level of AfaE expression in the S10EC strain compared with EC958 (*P < *0.0001). The amount of AfaE protein on the surface of the S10ECΔIS*1* mutant strain was 1.52-fold lower compared with wild-type S10EC (*P < *0.0001). No expression was detected in the S10ECΔ*afaE* and EC958Δ*afaE* mutant strains used as negative controls (Fig. 5B). As a final validation the IS*1* element was deleted in the promoter-*lacZ* fusion construct pQF50-Cm-*afa/dr*_S10EC and the promoter activity was measured by β-galactosidase assays. Deletion of IS*1* led to a 2-fold reduction (*P = *0.0028) in the promoter activity compared with the S10EC wild-type promoter; no β-galactosidase activity was identified in the strain carrying the empty plasmid pQF50-Cm (Fig. 5C). Taken together, these data provide direct evidence for the role of IS*1* in the increased expression of Afa adhesin in S10EC.

### The insertion of IS*1* modifies *afa/dr* regulation.

Afa/Dr fimbrial regulation is complex, involving H-NS (repression), IHF (activation), and Dam methylation (phase variation) (36–38). To test how integration of IS*1* affects the regulation of Afa, the *hns* and *ihfA* genes were deleted in EC958 and S10EC and the level of AfaE expression was analyzed by whole cell ELISA. EC958 displayed an expected regulatory phenotype, with deletion of *hns* causing increased AfaE expression (1.67-fold increase compared with wild-type EC958, *P = *0.014) and deletion of *ihfA* leading to decreased AfaE expression (2.4-fold decrease compared with wild-type EC958, *P = *0.0014) (Fig. S5A). In contrast, deletion of *hns* and *ihfA* in S10EC had no effect of AfaE expression (Fig. S5A). Phase variable expression of AfaE by EC958 and S10EC was measured by flow cytometry and immunofluorescence microscopy. EC958 existed as a heterogeneous population, with both phase-off and phase-on cells identified, and phase-off cells representing the majority of the population (Fig. S5B). In contrast, the population of S10EC was uniform and phase-on (Fig. S5C).

10.1128/mbio.03519-21.5 FIG S5 (A) ELISA showing the level of AfaE protein expressed on the surface of EC958 and S10EC wild-type and mutant derivatives. The primary polyclonal rabbit anti-AfaE-IX and the secondary alkaline phosphatase-conjugated anti-rabbit antibodies were used. Experiments were performed in three independent replicates. Unpaired student’s *t*-test was used for statistical analysis (* *P < *0.05; ** *P < *0.01; *** *P < *0.0001). (B) EC958 and (C) S10EC analysis of AfaE expression based on (i) flow cytometry and (ii) immunofluorescence microscopy. For flow cytometry analyses, the fluorescence intensity of EC958 and S10EC cells was measured using anti-AfaE-IX and secondary anti-rabbit AlexaFluor 488 antibodies. The blue lines represent the negative control (EC958Δ*afaE* and S10ECΔ*afaE*, respectively) used for setting the correct threshold value between noise and positive signal. The red lines represent the samples. The populations of EC958 phase-off, EC958 phase-on, and S10EC phase-on are indicated by arrows. For microscopy, phase contrast (left panels) and corresponding immunofluorescence (right panels) images are presented. Expression of the AfaE protein by EC958 and S10EC was detected primary polyclonal rabbit AfaE-IX antibody in combination with the secondary rabbit IgG FITC antibody. Download FIG S5, TIF file, 1.1 MB. Copyright © 2022 Alvarez-Fraga et al. 2022 Alvarez-Fraga et al. https://creativecommons.org/licenses/by/4.0/ This content is distributed under the terms of the Creative Commons Attribution 4.0 International license.

### Enhanced Afa/Dr fimbrial expression by S10EC leads to increased adherence to kidney epithelial cells.

**FIG 6:**
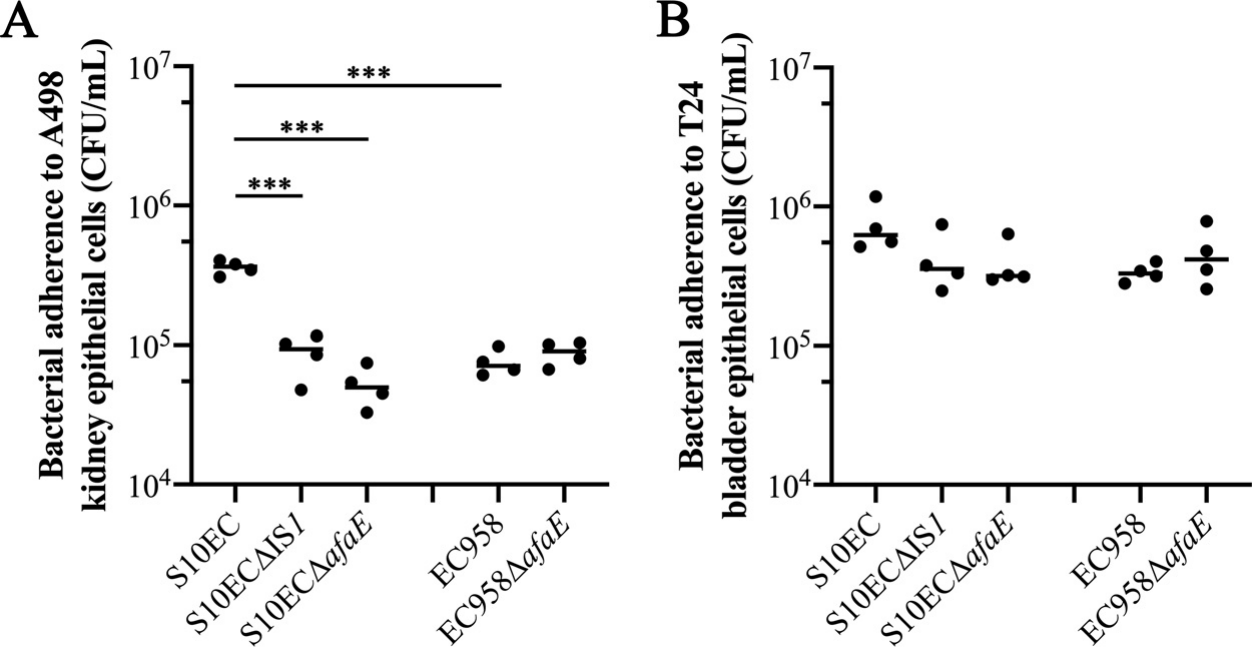
Adherence of S10EC, S10ECΔIS*1*, S10ECΔ*afaE*, EC958, and EC958Δ*afaE* strains to (A) A498 kidney and (B) T24 bladder human epithelial cells. Each data point is the mean of six technical replicates. Experiments were performed in four independent replicates. ANOVA and Šídák's multiple comparisons tests were used for statistical analysis (***, *P* value < 0.0001).

To determine the impact of IS*1*-mediated enhanced Afa/Dr fimbrial expression on virulence, we examined the capacity of our strains to adhere to and invade human kidney (A498) and bladder (T24) epithelial cells. This analysis revealed higher numbers of S10EC cells adhered to A498 kidney cells compared to EC958 (*P < *0.0001). When the IS*1* element or the *afaE* gene were deleted in S10EC, the adherence capacity was reduced to a level similar to EC958 (*P < *0.0001) (Fig. 6A). A similar trend was observed for adherence to T24 bladder epithelial cells, but the differences were not statistically significant (Fig. 6B). No significant difference was observed in the capacity of our strains to invade A498 kidney and T24 bladder epithelial cells (Fig. S6A, B). We also extended these analyses to test if increased Afa/Dr fimbrial expression enhanced colonization of the mouse bladder; however, no difference was observed in mixed competitive experiments using wild-type S10EC versus mutants deleted for IS*1* or Afa expression (Fig. S6C). Thus, IS*1*-mediated enhanced Afa/Dr expression leads to increased adherence to kidney cells but does not increase bladder cell adherence, epithelial cell invasion or bladder colonization under the conditions used in this study.

10.1128/mbio.03519-21.6 FIG S6 Invasion of S10EC, S10ECΔIS*1,* S10ECΔ*afaE*, EC958 and EC958Δ*afaE* strains to (A) A498 kidney and (B) T24 bladder human epithelial cells. Each data point is the mean of six technical replicates. Experiments were performed in three independent replicates. ANOVA and Šídák's multiple comparisons tests were used. (C) Mouse urinary tract colonization model. Competitive mixed-infection experiment employing a 1:1 mixture of E. coli S10EC/S10EC**Δ***lac,* E. coli S10ECΔIS*1*/S10ECΔ*lac,* E. coli S10ECΔ*afaC*/S10ECΔ*lac*, and E. coli S10ECΔ*afaC*/S10ECΔIS*1*Δ*lac.* Each symbol represents the log_10_ competitive index calculated for each mouse at 24-h postinfection. Horizontal bars represent median values. Statistical significance was determined by the two-tailed Wilcoxon’s signed rank test. Download FIG S6, TIF file, 0.5 MB. Copyright © 2022 Alvarez-Fraga et al. 2022 Alvarez-Fraga et al. https://creativecommons.org/licenses/by/4.0/ This content is distributed under the terms of the Creative Commons Attribution 4.0 International license.

### IS*1* contributes to genome evolution in ST131.

**FIG 7:**
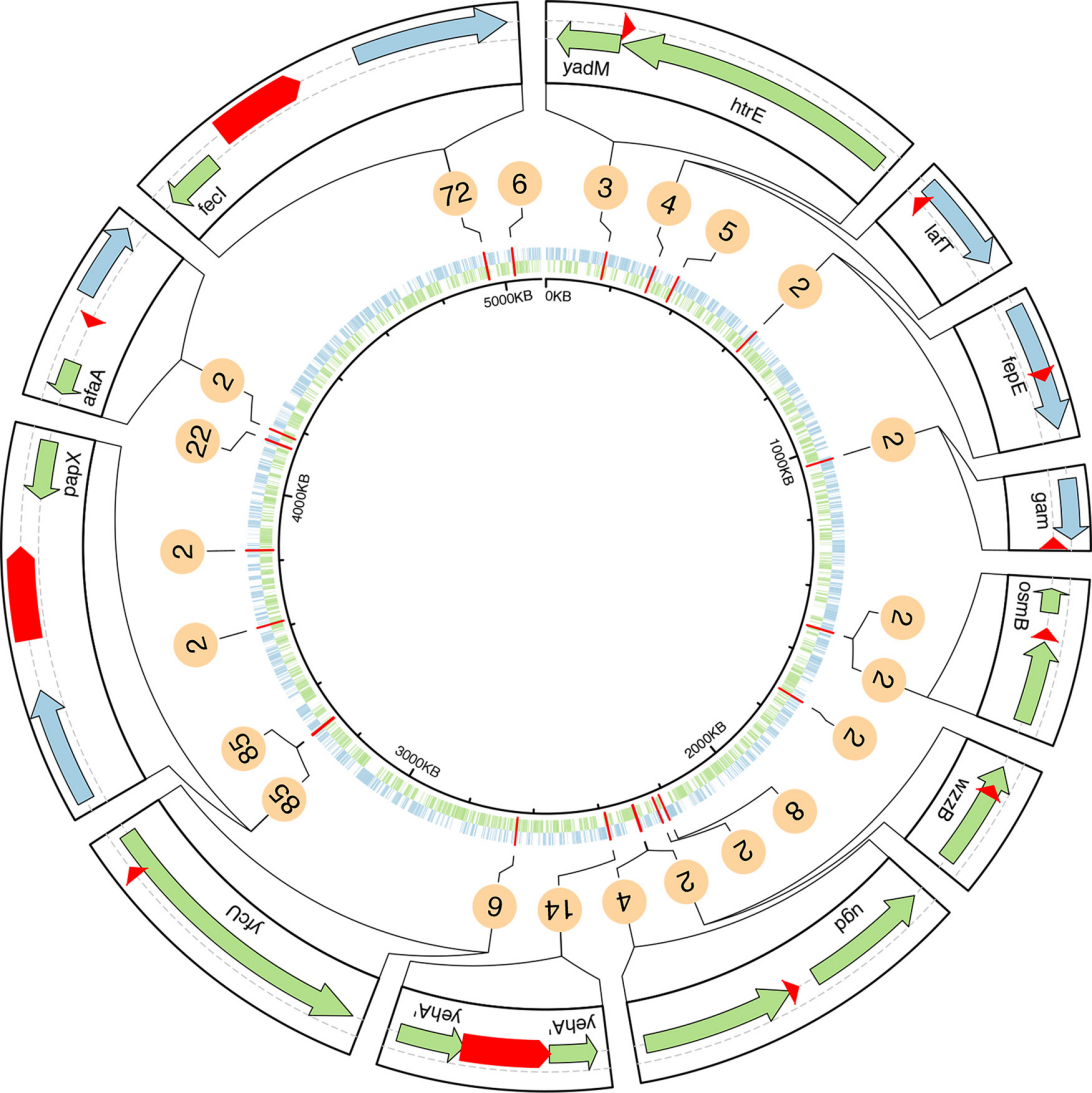
Circular genomic map showing the distribution of IS*1* elements within our 95 in-house ST131 strain collection (7). The black inner ring represents the E. coli EC958 reference genome. The distribution of minus strand ORFs (green) and plus strand ORFs (blue) are represented. The IS*1* elements are indicated in red and the number of strains possessing each IS*1* is represented in the circles. The outer ring represents 12 selected IS*1* elements with their respective flanking genes. Large red arrows represent IS*1* present in the EC958 reference strain. Small red arrows represent IS*1* not present in the EC958 strain. Arrows indicate orientation.

IS*1* is found abundantly in E. coli at both plasmid and chromosomal locations (51). The element comprises two adjacent transposase genes, *insA* and *insB*, flanked by short, inverted repeat sequences. To evaluate the impact of IS*1* on the evolution of ST131 at a chromosomal level, we examined its prevalence and location in our previously characterized set of 95 ST131 genomes, mapped against six completely sequenced reference genomes from this collection. Depending on the strain, we observed 113 to 125 IS*1* insertion sites relative to each corresponding reference genome, with a cumulative number of ∼160 unique insertion sites (Fig. S7A, Data set S1B and Data set S1C). We identified 124 insertion sites of IS*1* in our in-house ST131 strain collection relative to the EC958 reference genome (Data set S1B and Data set S1C). Among them, 22 were present in two or more strains at a prevalence of 2% to 90%; five insertions were found in EC958 (Fig. 7 and Data set S1D). To determine whether there was an association between IS*1* and large mobile genetic elements, we analyzed the location of each of the IS*1* identified in our set of 95 ST131 genomes using EC958 as the reference. The analysis revealed a correlation between IS*1* location and GIs, with the number of IS*1* insertion sites in GIs was significantly higher than the number of sites in the core genome (*P = *0.0013) (Fig. S7B). Overall, three different types of insertions were identified: (i) IS*1* inserted upstream of a coding sequence (CDS) in the same orientation, such as the *afaA*, *osmB*, or *ugd* genes; (ii) IS*1* inserted upstream of a CDS in the opposite orientation, such as *yadM*; and (iii) IS*1* inserted within a CDS, such as *yehA*, *wzzB*, or *fepE* (Fig. 7). To further investigate the impact of IS*1* when orientated in the same direction as a downstream gene, we examined the transcript levels of *afaA*, *osmB*, and *ugd* genes in EC958 (no insertion) compared with representative strains containing the IS*1* insertion. Consistent with our analyses described above, the transcript level of *afaA* was increased in strains that possess IS*1* compared to EC958 (∼ 7-log_2_ fold change, *P < *0.0001) (Fig. S8A). In contrast, the presence of IS*1* upstream of the *osmB* gene resulted in a reduction in the transcript level of this gene (∼ 2-log_2_ fold change, *P < *0.0001), while no difference was observed in the transcript level of the *ugd* gene (Fig. S8A). IS*1* contains an outward oriented −35 promoter box in its inverted repeat, and its insertion at the correct distance from a potential −10 box can generate a hybrid promoter (52). A close analysis of the IS*1* integration sites in the *afaA*, *osmB*. and *ugd* genes revealed the presence of a strong hybrid promoter generated by the insertion of IS*1* upstream of the *afaA* gene, but not the *osmB* and *ugd* genes (Fig. S8B).

10.1128/mbio.03519-21.7 FIG S7 (A) Plot showing the cumulative number of IS*1* insertion sites within the 95 in-house ST131 strain collection. Paired-end Illumina reads were mapped against six completely sequenced ST131 reference genomes (EC958, S10EC, S21EC, S22EC, S65EC, and S103EC). Each data point represents the number of insertion sites when different reference genomes were combined. (B) Plot showing the location of each of the IS*1* insertion sites within the 95 in-house ST131 strain collection. Paired-end Illumina reads were mapped against the EC958 reference genome. Each data point represents the number of IS*1* insertion sites found in each strain. Download FIG S7, TIF file, 0.4 MB. Copyright © 2022 Alvarez-Fraga et al. 2022 Alvarez-Fraga et al. https://creativecommons.org/licenses/by/4.0/ This content is distributed under the terms of the Creative Commons Attribution 4.0 International license.

10.1128/mbio.03519-21.8 FIG S8 (A) Transcript levels of the *afaA, osmB*, and *ugd* genes in the ST131 strains carrying an IS*1* element upstream of the gene. The results are represented as log_2_ fold change compared to EC958 (which has not insertion sequences). The *gapA* gene was used as an endogenous control. Experiments were performed in three biological replicates. Statistically significant differences are represented with asterisks (** *P* value < 0.01; *** *P* value < 0.0001). (B) Nucleotide sequence of the IS*1*-promoter region junctions. The right end of the IS*1* is underlined. The −35 region located in the IS*1* and the predicted −10 region are boxed. The asterisks show the nucleotides identical to the corresponding E. coli consensus sequences (−10, TATAAT; −35, TTGACA). Download FIG S8, TIF file, 0.9 MB. Copyright © 2022 Alvarez-Fraga et al. 2022 Alvarez-Fraga et al. https://creativecommons.org/licenses/by/4.0/ This content is distributed under the terms of the Creative Commons Attribution 4.0 International license.

## DISCUSSION

The Afa/Dr family form a group of fimbrial adhesins strongly associated with UPEC that cause gestational pyelonephritis and recurrent cystitis (16, 17, 53–57). Here, we have shown that the *afa/dr* genes occur frequently in strains from the globally disseminated multidrug resistant ST131 clone, and that the *afa/dr* promoter is a hotspot for IS integration, in some cases leading to increased expression associated with altered capacity for virulence.

The *afa* genes were found in 18/83 (21.7%) of the most common E. coli STs, spanning all phylogroups. The highest prevalence was observed in strains from ST131 and ST38, both of which represent important multidrug-resistant high-risk clones associated with UTI (3, 58–62). We also identified a different distribution of AfaE allelic variants within these two STs, consistent with their acquisition via independent horizontal gene transfer events. Our results are in line with previous studies using smaller strain collections that identified a significantly higher prevalence of *afa/dr* genes in ST131 compared with non-ST131 UPEC (4, 13). In the case of ST131, although we observed the predominance of a single previously undefined AfaE-IX allelic variant most closely related to NfaE-111, we speculate that it is unlikely the gain of *afa* fimbriae genes imparted a central fitness feature that drove its global dissemination. Rather, we suggest that Afa fimbriae modulate ST131 fitness, possibly by influencing colonization of the urinary tract. In this way, we predict that the expression of Afa fimbriae comprises one element of an intricate genetic landscape that contributes to the selective advantage and fitness of ST131. We note that Afa fimbriae also contribute to colonization of the gut by diffusely adhering E. coli (31), and therefore it is possible that the expression of Afa fimbriae by ST131 and ST38 strains increases intestinal colonization, thereby enabling a sustained reservoir for dissemination to extra-intestinal sites during infection as reported in a recent patient investigation (63).

Close analysis of the *afa/dr* cluster in ST131 revealed a hotspot for IS integration at a position 440-bp upstream of the *afaA* start codon. This corresponded to the insertion of IS*Cro1* or IS*Ec10* elements, as well as a 118-bp sequence of unknown origin, resulting in decreased *afaA* transcription and/or *afa* promoter activity. A further five ST131 strains, represented by S10EC, possessed an additional IS*1* element immediately downstream of IS*Cro1*, resulting in increased AfaE expression. The activation of gene transcription following the integration of an IS can occur in several ways, including the integration of a new promoter contained within the IS element (64–67) or the generation of a hybrid promoter formed by an outward oriented −35 promoter box at one end of the IS element proximal to a −10 box near the integration site (52, 68, 69). Multiple examples have been described in the literature in relation to antibiotic resistance; for example IS*1* integration upstream of the genes encoding the AcrEF efflux pump is associated with increased resistance in Salmonella enterica (70) and E. coli (71), and an IS*1*-like element is responsible for increased expression of the extended spectrum beta-lactamase TEM-6 in *Enterobacteriaceae* (72). Here, we describe a mechanism whereby IS*1* integration alters the regulation of a well-characterized virulence determinant, Afa/Dr fimbriae, leading to increased constitutive expression in some ST131 strains, and conferring an enhanced adherence phenotype. No significant difference was observed in the capacity of wild-type S10EC versus the mutant strains S10ECΔIS*1* and S10ECΔ*afaE* to invade uroepithelial cells or to colonize the mouse bladder in mixed competitive experiments. Although the mouse model of acute UTI has previously been used to demonstrate a role for Afa/Dr fimbriae in bladder colonization (40), we speculate that lack of a colonization defect for the S10EC mutants could be due to a difference in ligand specificity between the S10EC AfaE variant (AfaE-IX) compared to the VR50 AfaE variant (one amino acid difference compared to the AfaE-XI variant), both of which share only 17.3% amino acid identity (Fig. S3).

The contribution of IS to genome evolution and virulence is frequently overlooked. Previous studies of ST131 evolution have examined genome variation caused by single nucleotide polymorphisms without detailed investigation of the impact of IS elements (7, 10–12). Here we used a curated set of 95 ST131 genomes to map the spread of IS*1* locations, revealing ∼160 different IS*1* insertion sites in our genome data set. The integration of IS elements on the chromosome can impart a range of effects, including insertional inactivation of the target gene, the introduction of new cargo genes to the recipient strain, or altered transcription of adjacent genes (50, 73). Thus, in addition to altered regulation and expression of *afa/dr* genes caused by IS*1*, there are likely to be other changes associated with IS*1* integration that remain to be characterized. Indeed, comparison of IS*1* integration upstream of the *afaA*, *osmB*, and *ugd* genes revealed different outcomes; *afaA* transcription increased, *osmB* transcription decreased, and *ugd* transcription was largely unchanged. This reinforces our finding that although the integration of IS*1* can lead to the formation of a hybrid promoter that drives increased transcription of a downstream gene, this only occurs when the insertion is optimally located with respect to an adjacent −10 promoter element.

In summary, we have described the diversity and prevalence of *afa/dr* fimbrial genes in ST131, revealing the dominance of a new *afaE-IX* allelic variant. We additionally showed how different IS elements can alter the regulation of *afa* transcription and provide evidence for a more widespread impact of IS*1* on ST131 genome evolution. IS have been shown to play an important role in genome evolution of multiple pathogens, elegantly demonstrated by a recent study of *Shigella* species (74). Given the high carriage of IS*1* and other IS elements on antibiotic resistance plasmids found in ST131, our data support the contention that IS dynamics contribute to strain level microevolution that can affect multiple phenotypes, including resistance, virulence and metabolism.

## MATERIALS AND METHODS

Key experimental procedures used in the study are listed below. Extended experimental methods, including (i) whole genome sequencing and analysis, (ii) β-galactosidase assays, (iii) ELISA, (iv) flow cytometry, (v) immunofluorescence, (vi) epithelial cell adhesion and invasion assays, and (vii) mouse UTI model, are provided in Text S1 in the supplementary material.

10.1128/mbio.03519-21.9 TEXT S1 Extended experimental methods for (i) whole genome sequencing and analysis, (ii) β-galactosidase assays, (iii) ELISA, (iv) flow cytometry, (v) immunofluorescence, (vi) epithelial cell adhesion and invasion assays, and (vii) the mouse UTI model. Download Text S1, DOCX file, 0.03 MB. Copyright © 2022 Alvarez-Fraga et al. 2022 Alvarez-Fraga et al. https://creativecommons.org/licenses/by/4.0/ This content is distributed under the terms of the Creative Commons Attribution 4.0 International license.

### Bacterial strains and culture conditions.

All ST131 strains used in this study were previously described (7) and are listed in Table 1. Bacteria were grown routinely at 37°C in solid and liquid Luria-Bertani (LB) medium. Where appropriate, media was supplemented with 30 μg/mL of chloramphenicol (Cm) and/or 20 μg/mL of gentamicin (Gm).

### Bioinformatic analysis.

Assemblies of E. coli strains belonging to ST10, ST69, ST73, ST95, and ST131 were downloaded from EnteroBase in July 2018, resulting in a collection of 11,283 strains (https://enterobase.warwick.ac.uk) (44). Assemblies of E. coli strains belonging to ST38 were downloaded from EnteroBase in November 2020, resulting in a collection of 1,926 strains. In addition, approximately 100 sequence assemblies were randomly chosen from each of the top 83 E. coli STs in the E. coli collection from EnteroBase (8,247 strains). ST131 strains were classified in clades and subclades based on clade-specific SNPs (10). The prevalence of Afa/Dr fimbriae was determined using the BLAST software package and the *afaC* gene from EC958 (75). The cut-off values used for filtering were > 90% nucleotide identity and > 80% gene coverage. The distribution of each *afaE* allele in ST38 and ST131 and the *afa/dr* promoter region in ST131 was analyzed using the BLAST software package. The cut-off values used for filtering were > 97% nucleotide identity and > 80% gene coverage. Analysis of BLAST output files analysis was carried out in R (version 3.6.2) through the R Studio environment (version 1.1.442) using the tidyverse package (version 1.2.1). DNA sequences were visualized using Artemis version 18.1.0 and CLC Main Workbench v8.1.3. The genomic context of genes was analyzed and drawn with Easyfig (76). Protein sequence alignments were performed with ClustalO and phylogenetic trees were generated using IQ-TREE v.1.6.8 and visualized and edited using FigTree v1.4.4. Paired-end Illumina reads from 95 ST131 strains were examined using ISmapper (77) to identify IS*1* insertion sites relative to each of the six completely sequenced ST131 reference genomes (EC958, S10EC, S21EC, S22EC, S65EC, and S103EC). To estimate the number of IS*1* insertion sites in multiple reference genomes, each insertion site was considered the same when sharing the same flanking genes with the same distances to the left and right genes. The R package circlize (version 0.4.11) was used to visualize the insertion sites of IS*1* relative to the EC958 reference genome (HG941718).

### RNA extraction and quantitative reverse transcription-PCR (qRT-PCR).

Bacteria were grown to exponential phase (optical density [OD] at 600 nm = 0.6) and stabilized with 2 volumes of RNAprotect bacterial reagent (Qiagen). Total RNA was isolated using RNeasy minikit (Qiagen) following the manufacturer's instructions. RNA samples were treated with RNase-free DNase I to remove contaminating DNA and purified using Qiagen RNeasy columns. cDNA was synthesized using the SuperScript®III First Strand Synthesis System (Life Technologies) according to the manufacturer's instructions. Real-time PCR (qPCR) reactions were performed using SYBR Green Master Mix (Applied Biosystems) on a QuantStudio 6 instrument (Applied Biosystems). The transcript level of each tested gene was normalized relative to the transcript level of the housekeeping gene *gapA*. All experiments were performed as three independent replicates. Statistical analysis was performed using an unpaired, two-tailed Student's *t* test. Primers used are listed in Data set S1E.

### Plasmid construction.

Molecular methods were performed according to standard protocols as previously described (78). The *afa/dr* promoter-*lacZ* fusion constructs were created by PCR amplification of the different *afa/dr* promoter regions and cloned in front of the promoterless *lacZ* gene in the plasmid pQF50-Cm via NcoI-BamHI or SalI-BamHI digestion (see Data set S1E for primer list). The resulting plasmids were transformed into the EC958Δl*ac* mutant strain. Transformants were selected following growth on LB agar containing chloramphenicol and checked by PCR followed by sequencing.

### Mutant construction.

All mutants were generated using the λ-Red recombinase gene replacement system (79). Briefly, a three-way PCR procedure was performed to amplify the chloramphenicol cassette from plasmid pKD3 with ∼500 bp homologous arms flanking the region targeted for deletion. The fused PCR products were electroporated into the wild-type strains harboring the gentamicin resistant plasmid pKOBEG-G carrying the λ-Red recombinase gene. When necessary, the chloramphenicol resistance cassette was removed using plasmid pCP20-G (80). Mutants were confirmed by PCR followed by sequencing using the primers listed in Data set S1E.

### Western blotting.

Whole cell lysates were prepared by pelleting 1 mL of OD_600_ = 1.0 standardized cell suspensions and resuspending the cells in 50 μL water and 50 μL of 2X loading buffer (2X NuPAGE LDS Sample Buffer, 200 mM DTT) and heating the samples at 100°C for 5 min. SDS-PAGE and Western blotting was performed as previously described (81). Rabbit polyclonal antisera specific for AfaE-IX (1:200) and OmpA (1:50,000) were used as primary antibodies and alkaline phosphatase-conjugated anti-rabbit antisera (Sigma-Aldrich) (1:15,000) was used as the secondary antibody. Blots were developed using BCIP/NBT stock solution.

### Epithelial cell assays.

Afa/Dr fimbriae-mediated adherence and invasion was assessed using human T24 bladder (ATCC HTB-4) and A498 kidney (ATCC HTB-44) epithelial cells. T24 cells express the Afa fimbriae target DAF receptor (82). While DAF expression has not been directly demonstrated for A498 cells, it is well-established that the DAF receptor is expressed by human kidney cells (83). Full methods are provided in Text S1 in the supplementary material.
