# Supplementary figures and images for: Differential Afa/Dr Fimbriae Expression in the Multidrug-Resistant Escherichia coli ST131 Clone
Source: mBio. 2022 Jan 18;13(1):e03519-21. doi: 10.1128/mbio.03519-21 (PMC8764528; doi:10.1128/mbio.03519-21)

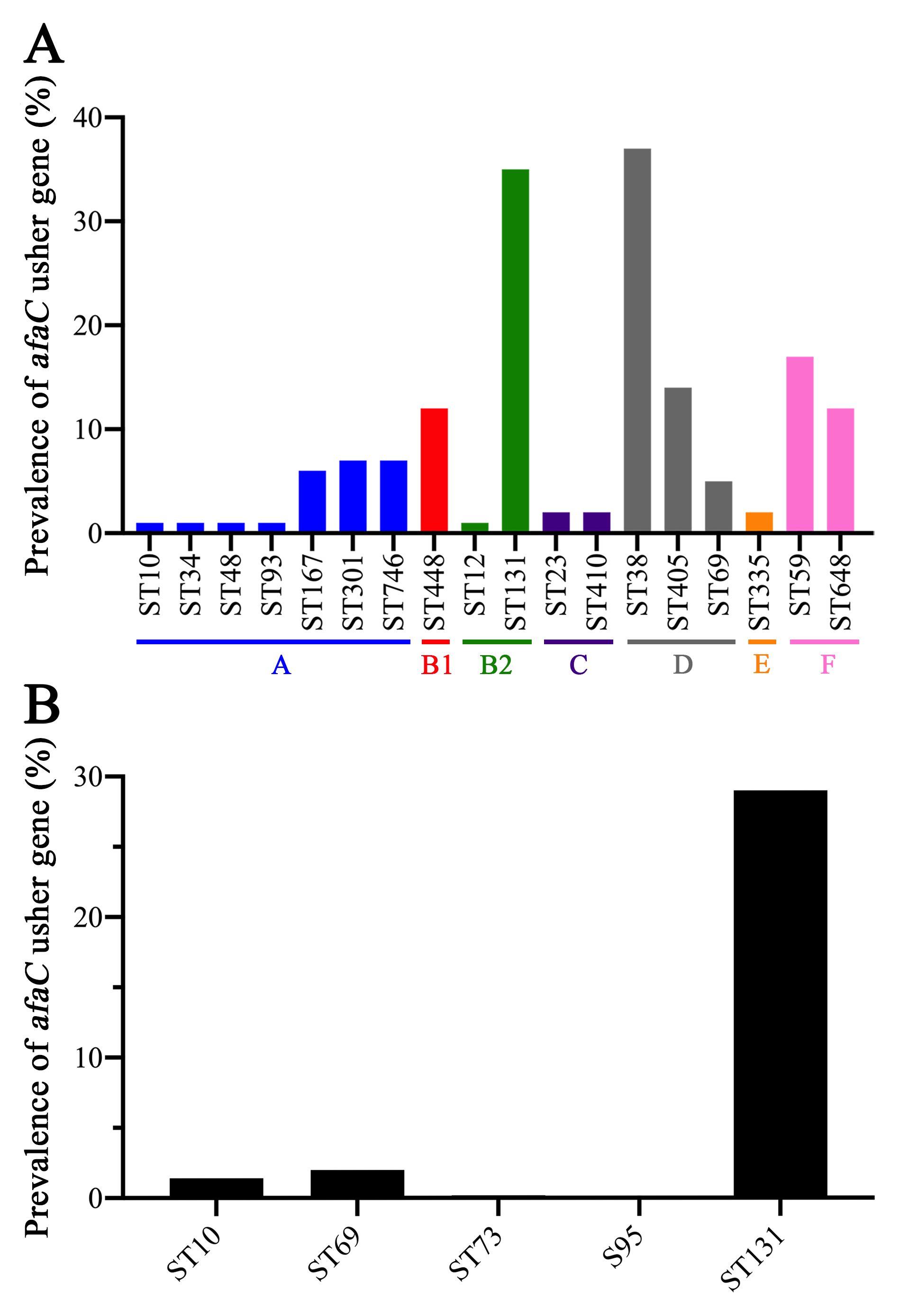

Supplement: FIG S1 [file mbio.03519-21-sf001.tif]

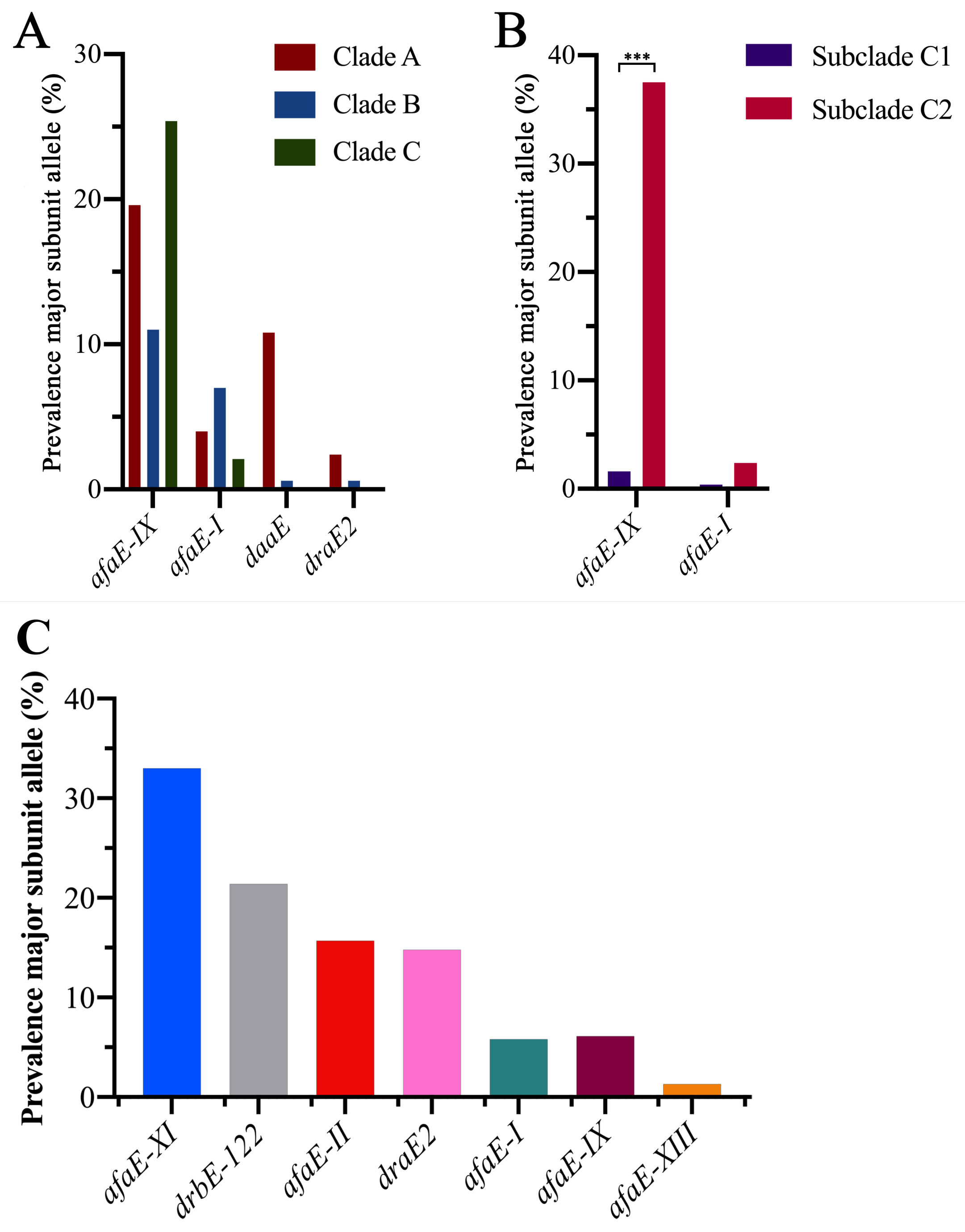

Supplement: FIG S2 [file mbio.03519-21-sf002.tif]

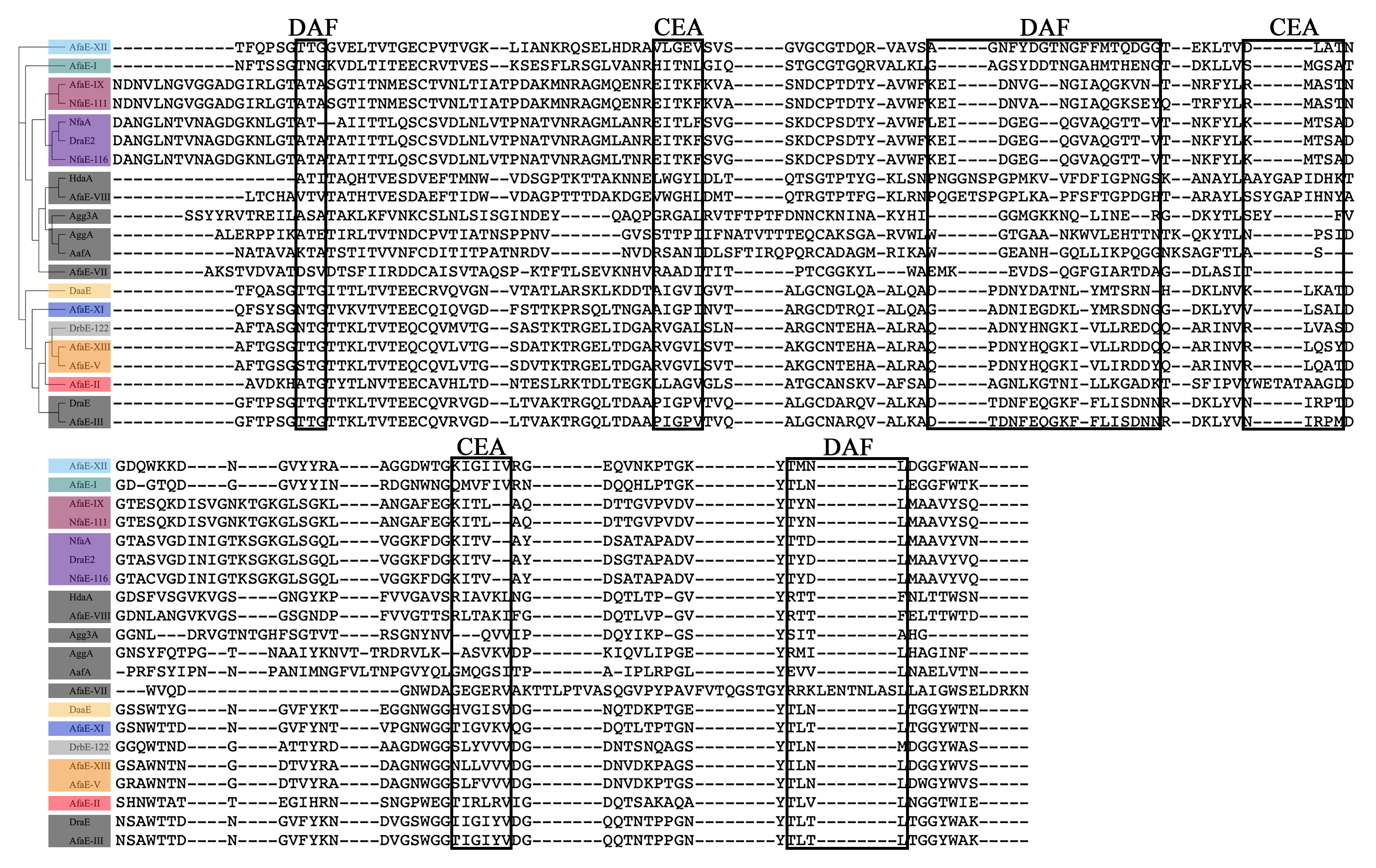

Supplement: FIG S3 [file mbio.03519-21-sf003.tif]

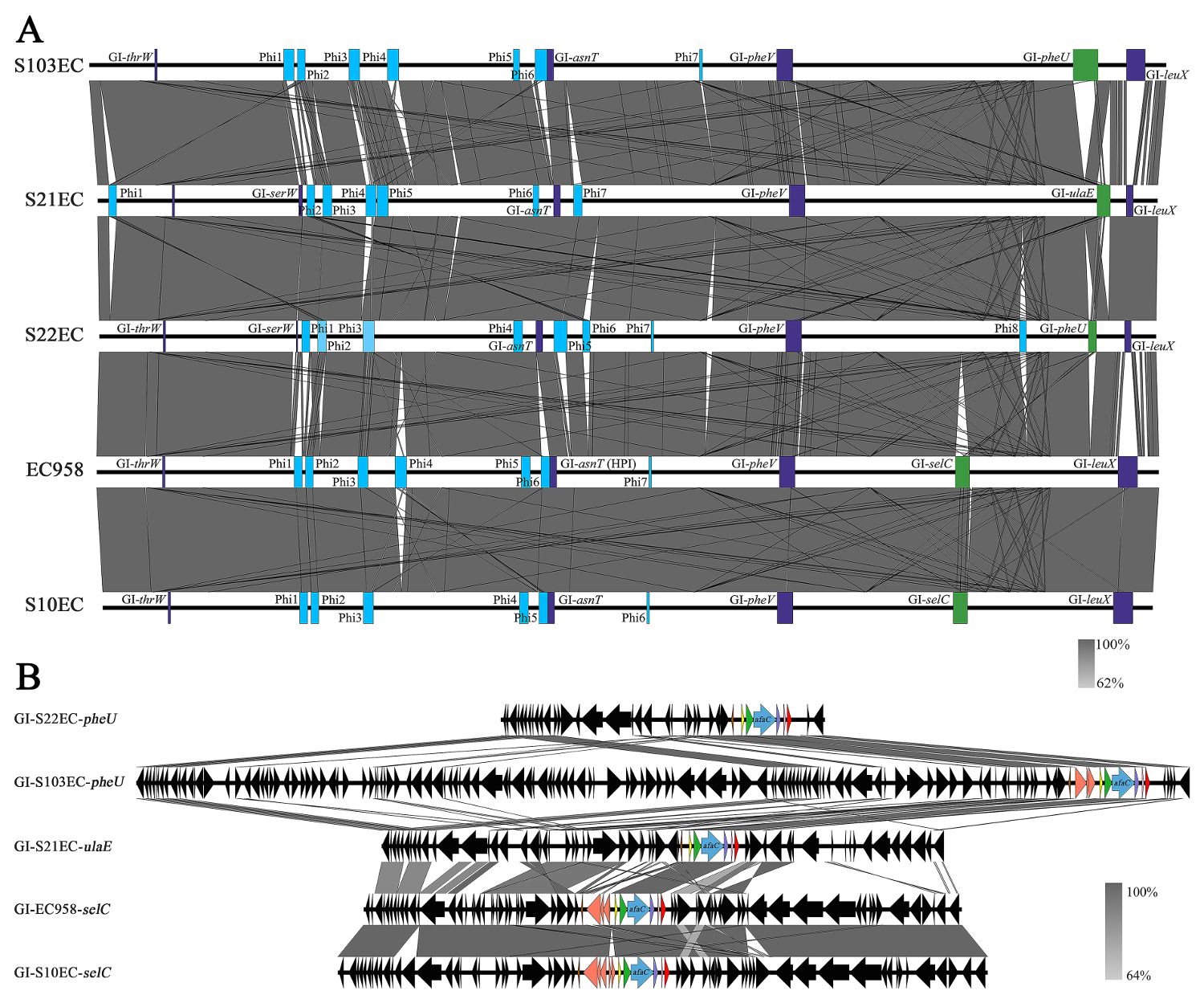

Supplement: FIG S4 [file mbio.03519-21-sf004.tif]

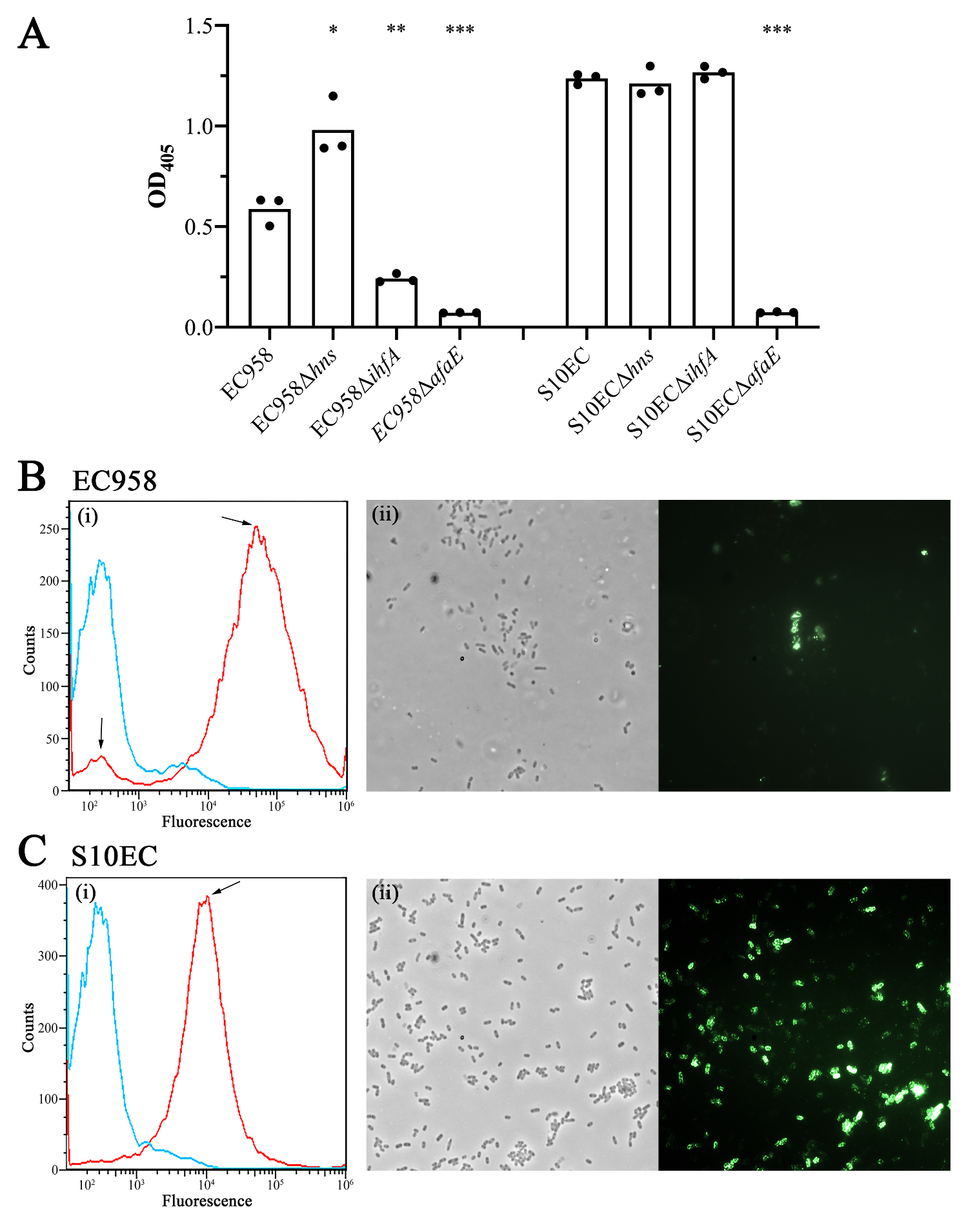

Supplement: FIG S5 [file mbio.03519-21-sf005.tif]

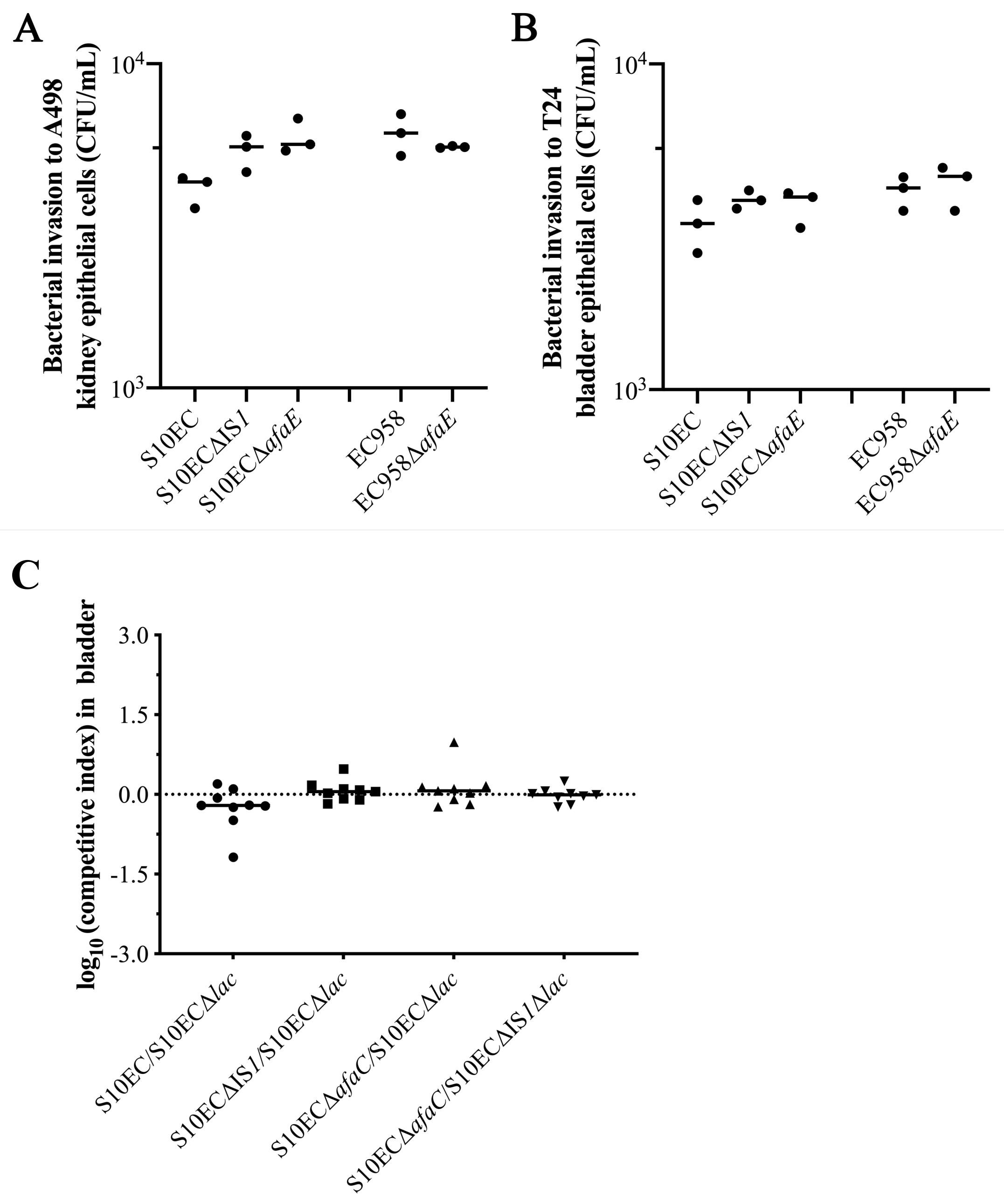

Supplement: FIG S6 [file mbio.03519-21-sf006.tif]

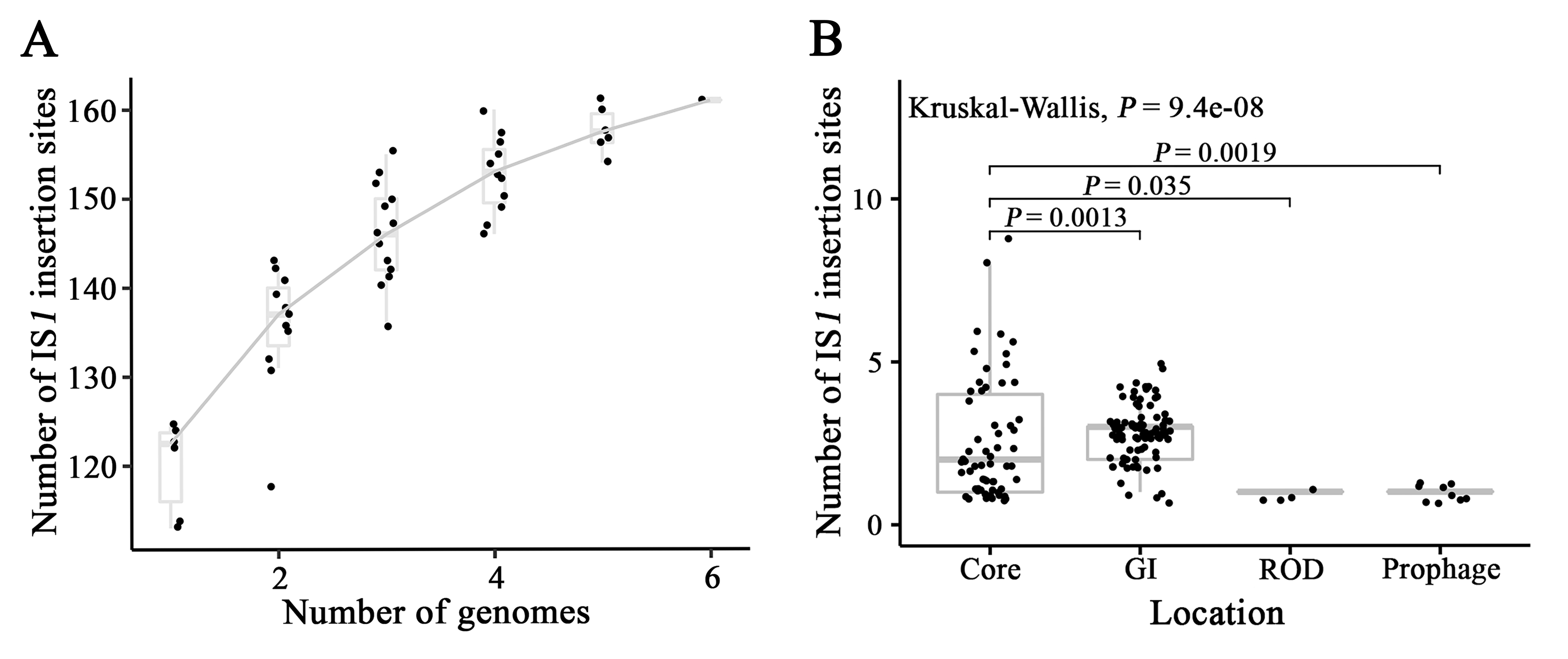

Supplement: FIG S7 [file mbio.03519-21-sf007.tif]

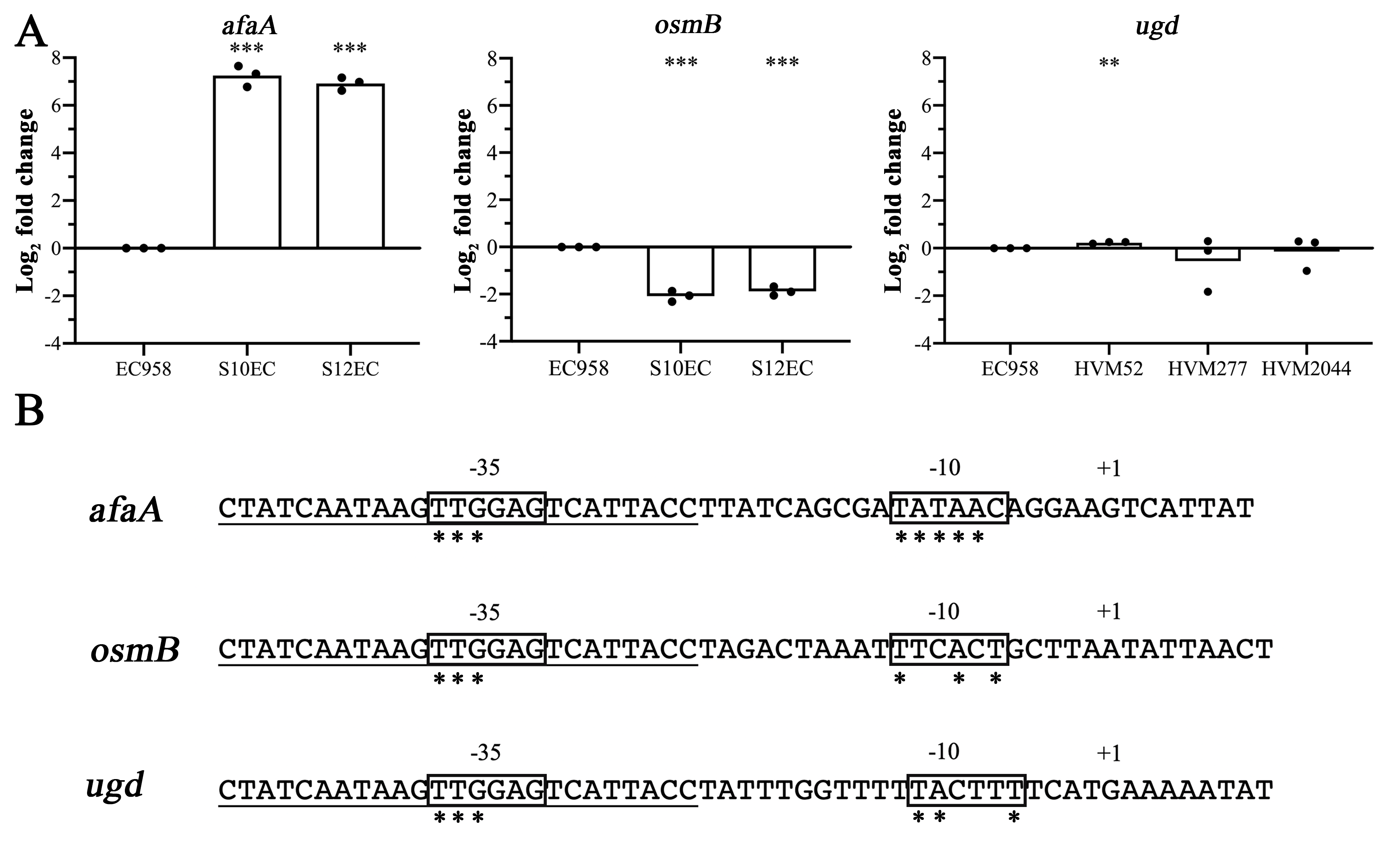

Supplement: FIG S8 [file mbio.03519-21-sf008.tif]
