# Supplementary material for: Differential Afa/Dr Fimbriae Expression in the Multidrug-Resistant Escherichia coli ST131 Clone
Source: mBio. 2022 Jan 18;13(1):e03519-21. doi: 10.1128/mbio.03519-21 (PMC8764528; doi:10.1128/mbio.03519-21)
Supplement: TEXT S1 [file mbio.03519-21-t0001.docx]

**SUPPLEMENTARY MATERIAL AND METHODS**

(i) Whole genome sequencing and assembly

*E. coli* isolates (S10EC, S103EC, S21EC and S22EC) were grown from single colonies in Lysogeny Broth (LB) at 37ºC overnight with 250 rpm shaking. The overnight cultures (1.5 mL) were then pelleted for DNA extraction using the Wizard Genomic DNA Purification Kit (Promega) following manufacturer’s protocol with modifications. Briefly, the cell pellet was lysed following the protocol for Gram negative bacteria. RNA was removed by 1h incubation at 37°C with RNase and the lysate was then mix with Protein Precipitation Solution by vortexing for 5s at max speed using Vortex-Genie 2 with horizontal tube adapter (Scientific Industries). The DNA was precipitated using isopropanol and washed with 70% ethanol. The DNA pellet was air-dried and then rehydrated in 100 µl EB buffer (QIAgen) by incubation at 65°C for 1 hour. The DNA was quantified using a Qubit fluorometer (ThermoFisher Scientific) and the DNA fragment size (>50kb) was estimated using agarose gel electrophoresis (0.5% agarose in TAE, 90V, 1h30m). The DNA from these isolates (~800 ng each) were multiplexed (to a total of 12 isolates) onto a single FLO-MIN106 flow cell using the rapid barcode sequencing kit (SQK-RBK004) as per manufacturer’s recommendation with the following adjustments: the barcoded DNA was pooled without a concentration step using AMPure XP beads prior to sequencing. Nanopore basecalling and assembly of these genomes were performed using MicroPIPE v0.9.1 (1), which used guppy base caller (guppy-gpu v4.4.1), qcat (v1.0.1) for demultiplexing, porechop for adapter trimming (v0.2.3), jaspa (v1.9-10b) for read filtering, flye (v2.5) for long read assembly, Racon (v1.4.9)/Medaka (v0.10.0) for long read polishing, nextpolish (v1.1.0) for short read polishing, circlator (v1.5.5) for fixing start position, and quast (5.0.2) for assembly statistics. The Illumina reads used for short read polishing were downloaded from EBI using the following accession numbers: ERR161237, ERR161266, ERR161242 and ERR161243 and trimmed using trimmomatics (v0.36). The genome assemblies were uploaded to NCBI under the BioProject no. PRJNA737575. The genome accession numbers are listed in Dataset S1A.

(ii) β-galactosidase assays

β-galactosidase assays were performed as previously described (2). Briefly, EC958Δ*lac* carrying *lacZ* fusions were grown on LB-Cm plates for 16 h then inoculated into LB-Cm medium. After 16-18 h of growth, 75 µL of the bacterial cultures were diluted in Z-buffer (60 mM Na_2_HPO_4_, 40 mM NaH_2_PO_4_, 50 mM β-mercaptoethanol, 10 mM KCl, 1 mM MgSO_4_, pH 7)-0.1% SDS and chloroform and vortexed to permeabilize the cells. Samples were incubated at 28°C for 1 hour and the reaction was initiated by the addition of ortho-Nitrophenyl-β-galactoside (ON­PG). Reactions were stopped with the addition of sodium bicarbonate and the enzymatic activity was assayed by measuring the absorbance at 420 nm. All experiments were performed in three independent replicates. Statistical analysis of β-galactosidase levels was performed using an unpaired, two tailed student’s t-test.

**(iii) Enzyme-linked immunosorbent assay (ELISA)**

Bacterial suspensions were prepared by standardizing overnight cultures to OD_600_ = 0.6 in carbonate coating buffer. For each sample, a volume of 100 µL per was added to six different wells in a 96-well microtiter plate MaxiSorp (Nunc) and incubated overnight at 4°C statically. The samples were blocked with 2% skim milk in phosphate buffered saline-Tween 20 (T-PBS) for 1 hour at room temperature and incubated with anti-AfaE-IX (1:200) or anti-*E. coli* (Meridian Bioscience) (1:10000) antibodies for 1 hour at room temperature. Alkaline phosphatase-conjugated anti-rabbit antibody (Sigma-Aldrich) (1:15000) was added to the samples and incubated for 1 hour at room temperature. Development was performed by adding alkaline phosphatase yellow (pNPP) liquid substrate (Sigma-Aldrich) and reading the OD_405_ after 20 minutes. All experiments were performed as three independent replicates with six technical replicates. Statistical analysis was performed using an unpaired, two-tailed student’s t-test.

(iv) Flow cytometry

Bacterial overnight cultures were standardized to an optical density at 600 (OD_600_) of 0.6 in 1X PBS and 1 mL of each strain was pelleted and resuspended in 50 μl of the rabbit polyclonal anti-AfaE-IX (1:50). Cells were incubated for 30 minutes at room temperature with the primary antibody, washed 3 times and incubated another 30 minutes in the dark with the secondary antibody Goat anti-rabbit AlexaFluor 488 conjugate (1:500). Cells were washed 3 times and resuspended in 0.1% formalin. The Accuri C6 (BD Biosciences) flow cytometer was used for analysing the samples. Negative controls were included in order to set the correct threshold value between noise and positive signal.

(v) Immunofluorescence microscopy

Cells grown overnight in LB agar were harvested, washed and standardized to OD_600_ 0.6 in MQ water. Thirty μL of each standardized bacterial suspension were spotted on duplicate wells on a multi spot slide, air-dried and fixed with 4% solution of paraformaldehyde in 1X PBS for 10 minutes at room temperature. Cells were washed three times to remove the fixation solution and incubated 3 minutes with 50mM of NH_4_Cl. Cells were incubated 15 minutes in 0.5% bovine serum albumin (BSA) and incubated 30 minutes with the rabbit polyclonal anti-AfaE-IX primary antibody (1:50) at room temperature. The slides were washed four times with BSA/PBS and incubated with the anti-rabbit IgG FITC secondary antibody (1:100). After 30 minutes in the dark, the slides were washed four times with BSA/PBS, dried and mounted with ProLong Gold. An epifluorescence microscope (Axioplan 2; Zeiss) equipped with detectors and filters for monitoring fluorescein isothiocyanate (FITC) was used to visualize the samples.

**(vi) Epithelial cell adhesion and invasion assays**

T24 bladder (ATCC HTB-4) and A498 kidney (ATCC HTB-44) epithelial cells were routinely grown at 37°C with 5% CO2 in complete RPMI (cRPMI) medium (RPMI 1640 supplemented with 25 mM HEPES, 2 mM L-glutamine, 10% heat inactivated fetal bovine serum, 100 mM nonessential amino acids, 1 mM sodium pyruvate, 100U/ml penicillin, and 100 mg/ml streptomycin). For both adhesion and invasion assays, 5 x 10^4^ cells were seeded into a 96-well tissue culture plate and incubated overnight at 37°C with 5% CO2. The next day, the monolayers were infected with 1 x 10^6^ bacteria (multiplicity of infection [MOI] of 10) and incubated at 37°C. After 1h, unbound bacteria were removed by washing with PBS. For adhesion assays, the monolayers were lysed by the addition of a 0.02% Triton X-100 and 0.05% trypsin/EDTA solution. For invasion assays, the monolayers were first incubated at 37°C for an additional 1h in the presence of cRPMI + 250U/ml penicillin, 250 mg/ml streptomycin and 50μg/ml of gentamicin to kill extracellular bacteria before lysis. The number of adherent or intracellular bacteria was determined by serial dilution and plating on LB agar. All assays were performed in the presence of 1% methyl α-D-mannopyranoside to negate the impact of type 1 fimbriae.

(vii) Mouse model of UTI

The C57BL/6 mouse model of ascending UTI was employed as previously described (3). All strains used in this experiment were enriched for type 1 fimbriae expression after 3 passages under static conditions (4). Bacterial strains were grown statically overnight in LB broth and type 1 pili expression was confirm by yeast cell agglutination and the *fim* switch orientation PCR (5). Infections were performed as competitive assays with inoculum containing 1:1 strain mixture. An inoculum of 20–40 μl, containing 5×10^8^ CFU of bacteria in PBS was injected directly into the bladder of each mouse (n=8). Bacterial loads corresponding in the urine, bladder, and kidney at 24 hours post-infection were enumerated by plating onto MacConkey agar, which allowed the differentiation of *lac* mutant (non-lactose fermenter) from the wild type strains. Statistical differences were determined using the two-tailed Wilcoxon matched pairs test.

**REFERENCES**

1. Murigneux V, Roberts LW, Forde BM, Phan M-D, Nhu NTK, Irwin AD, Harris PNA, Paterson DL, Schembri MA, Whiley DM, Beatson SA. 2021. MicroPIPE: <em>An end-to-end solution for high-quality complete bacterial genome construction</em>. bioRxiv doi:10.1101/2021.02.02.429319:2021.02.02.429319.

2. Tan L, Moriel DG, Totsika M, Beatson SA, Schembri MA. 2016. Differential Regulation of the Surface-Exposed and Secreted SslE Lipoprotein in Extraintestinal Pathogenic Escherichia coli. PLoS One 11:e0162391.

3. Roos V, Ulett GC, Schembri MA, Klemm P. 2006. The asymptomatic bacteriuria Escherichia coli strain 83972 outcompetes uropathogenic E. coli strains in human urine. Infect Immun 74:615-24.

4. Totsika M, Beatson SA, Sarkar S, Phan MD, Petty NK, Bachmann N, Szubert M, Sidjabat HE, Paterson DL, Upton M, Schembri MA. 2011. Insights into a multidrug resistant Escherichia coli pathogen of the globally disseminated ST131 lineage: genome analysis and virulence mechanisms. PLoS One 6:e26578.

5. Schembri MA, Olsen PB, Klemm P. 1998. Orientation-dependent enhancement by H-NS of the activity of the type 1 fimbrial phase switch promoter in Escherichia coli. Mol Gen Genet 259:336-44.
